# Interferon-inducible protein, IFIX, has tumor-suppressive effects in oral squamous cell carcinoma

**DOI:** 10.1038/s41598-021-99157-4

**Published:** 2021-10-01

**Authors:** Shan Wang, Fang Li, Haixia Fan

**Affiliations:** 1 Department of Oral Pathology, Hospital of Stomatology, The First Affiliated Hospital, Harbin Medical University, Harbin, 150001 People’s Republic of China; 2 Institute of oral biomedicine, Heilongjiang Academy of Medical Science, Harbin, 150086 People’s Republic of China; 3 Department of Oral and Maxillofacial Surgery, Hainan Maternal and Children’s Medical Center, Haikou, 570000 People’s Republic of China; 4 Department of Oral Medicine, Jining Medical College, Jining, 272067 People’s Republic of China

**Keywords:** Cancer, Biomarkers, Medical research, Oncology

## Abstract

IFIX, a newly discovered member of the interferon-inducible HIN-200 family, has been identified as a tumor suppressor in breast cancer; however, the involvement of IFIX in oral cancer are poorly understood. Here, we demonstrate a relationship between the level of IFIX expression and the invasive or migratory abilities of oral squamous cell carcinoma. Higher IFIX expression significantly correlated with clinicopathological parameters such as the histopathological grade of clinical samples. In vitro, IFIX overexpression suppressed the invasiveness of human tongue squamous cell carcinoma CAL-27 cells, and this inhibitory effect was mediated by stabilization of the cytoskeleton through various cytokeratins along with downregulation of paxillin, an intracellular adaptor protein that promotes tumor invasion. This inhibitory effect does not appear to affect the transformation of cancer stem-like cells in this cell culture model. Altogether, these data provide novel insights into the tumor-suppressive function of IFIX, namely, stabilization of the cancer cell cytoskeleton.

## Introduction

Head and neck squamous cell carcinoma (HNSCC), including oral cancer, is the sixth most common malignancy^1^, and oral squamous cell carcinoma (OSCC) is the most common subtype of HNSCC^2^. In China, the incidence of oral and oropharyngeal cancers fluctuated from 2005 to 2013, whereas the mortality rate showed an upward trend after 2009. A heavier burden of oral and oropharyngeal cancers has been predicted in the next two decades in China^3^. The prognosis of OSCC is still unfavorable due to aggressive local invasion and metastasis at clinical stage TNM I/II, resulting in high recurrence^4,5^. Therefore, the detection and treatment of early metastasis are key determinants of OSCC prognosis.

The PYRIN and HIN domain (PYHIN) family consists of five interferon-inducible proteins that were recently identified as intracellular DNA sensors. These include interferon-inducible protein 16 (IFI16), absent in melanoma 2 (AIM2), myeloid cell nuclear differentiation antigen (MNDA), pyrin and HIN domain family 1 (PYHIN1 or IFIX), and PYRIN domain (PYD)-only protein 3 (POP3)^6–8^. AIM2 is a member of the HIN-200 family and has been previously implicated in OSCC. Nakamura et al. found that high AIM2 expression in OSCC causes cancer progression, thereby promoting epithelial–mesenchymal transition (EMT)^9^. Another study on colorectal cancer carcinogenesis, which shares similarities with OSCC in epidemiological patterns, has illustrated that AIM2 deficiency contributes to the maintenance of intestinal stem cells that are prone to uncontrolled proliferation^10^.

Of note, IFIX is the most recent member of the PYHIN family to be identified^11,12^, and its functions have been linked to tumor suppression, as evidenced by its antitumor and antiproliferative activities and its downregulation in human breast tumors and breast cancer cell lines^11^. IFIX has been shown to destabilize human double minute homolog (HDM2), which in turn leads to increased p53 levels. Stabilization of p53 by IFIX may indicate the mechanism by which IFIX exerts its tumor-suppressive action^13^.

The purpose of this study was to explore the function of IFIX in OSCC cells by determining the relationship between IFIX expression and OSCC invasiveness or migration. The results suggest that higher IFIX expression significantly correlates with clinicopathological parameters, such as histopathological grade. Higher IFIX expression in OSCC cells resulted in decreased migration and invasiveness by stabilizing the cytoskeleton through components such as cytokeratins, and by downregulating paxillin, which is closely associated with cell migration and survival. These data may implicate an inhibitory mechanism in EMT. In contrast, this phenomenon does not seem to be significantly or directly related to the transformation of cancer stem-like cells (CSCs) outside of the tumor microenvironment.

## Results

### IFIX expression in OSCC and its precursor stages

**Figure 1:**
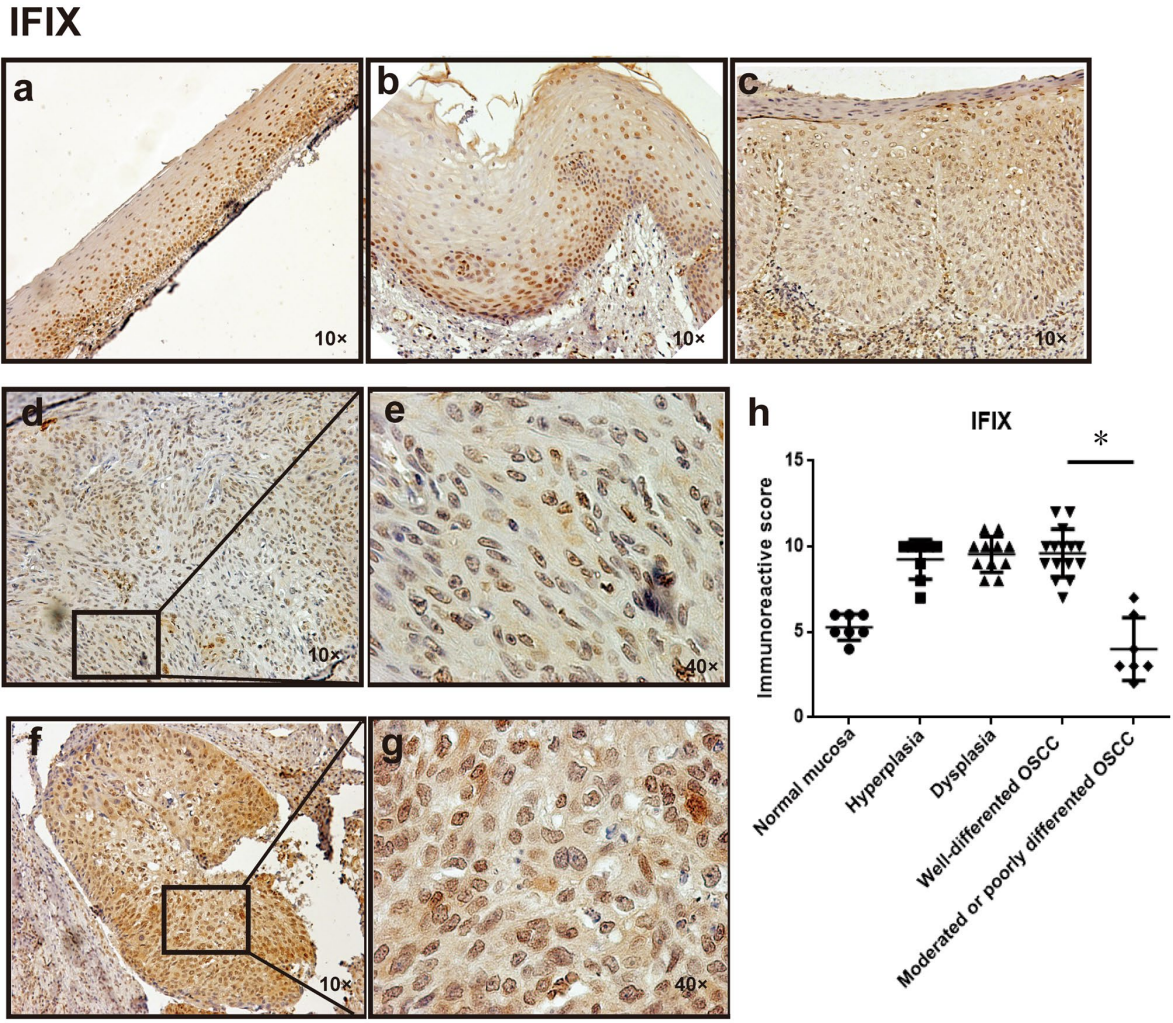
Characteristic IFIX expression in OSCC and its precursor stages. (**a**–**c**) IFIX expression by IHC in histopathologically assessed normal mucosa, hyperplasia, and dysplasia. (**d**,**e**) Lower expression of IFIX in moderately differentiated OSCC. (**f**,**g**) Higher expression of IFIX in well-differentiated OSCC cells. (**h**) The pattern of immunoreactive scores of IFIX staining in cancer tissue samples of OSCC at various histopathological grades and OSCC precursor stages.

To explore IFIX expression throughout oral cancer development, we first measured IFIX distribution by immunohistochemistry (IHC) in formalin-fixed paraffin-embedded samples derived from oral lesions. IFIX was stably expressed in both normal oral mucosa and OSCC tissues. Of note, IFIX was mainly expressed in the nuclei of cells in the stratum basale and stratum spinosum in oral mucosa or hyperplasia tissue (Fig. 1a,b). In contrast, at the oral mucosal mild dysplasia stage, or formed cancer status, IFIX was detected in the cytoplasm and nucleus (Fig. 1c–g). Furthermore, well-differentiated OSCC tissue showed higher IFIX expression (Fig. 1f,g) in comparison to moderately or poorly differentiated OSCC tissue (Fig. 1d,e). There was a significant difference in the immunoreactive score between well-differentiated OSCC tissue samples and moderately or poorly differentiated OSCC (Fig. 1h).

### IFIX overexpression exerts tumor-suppressive effects

**Figure 2:**
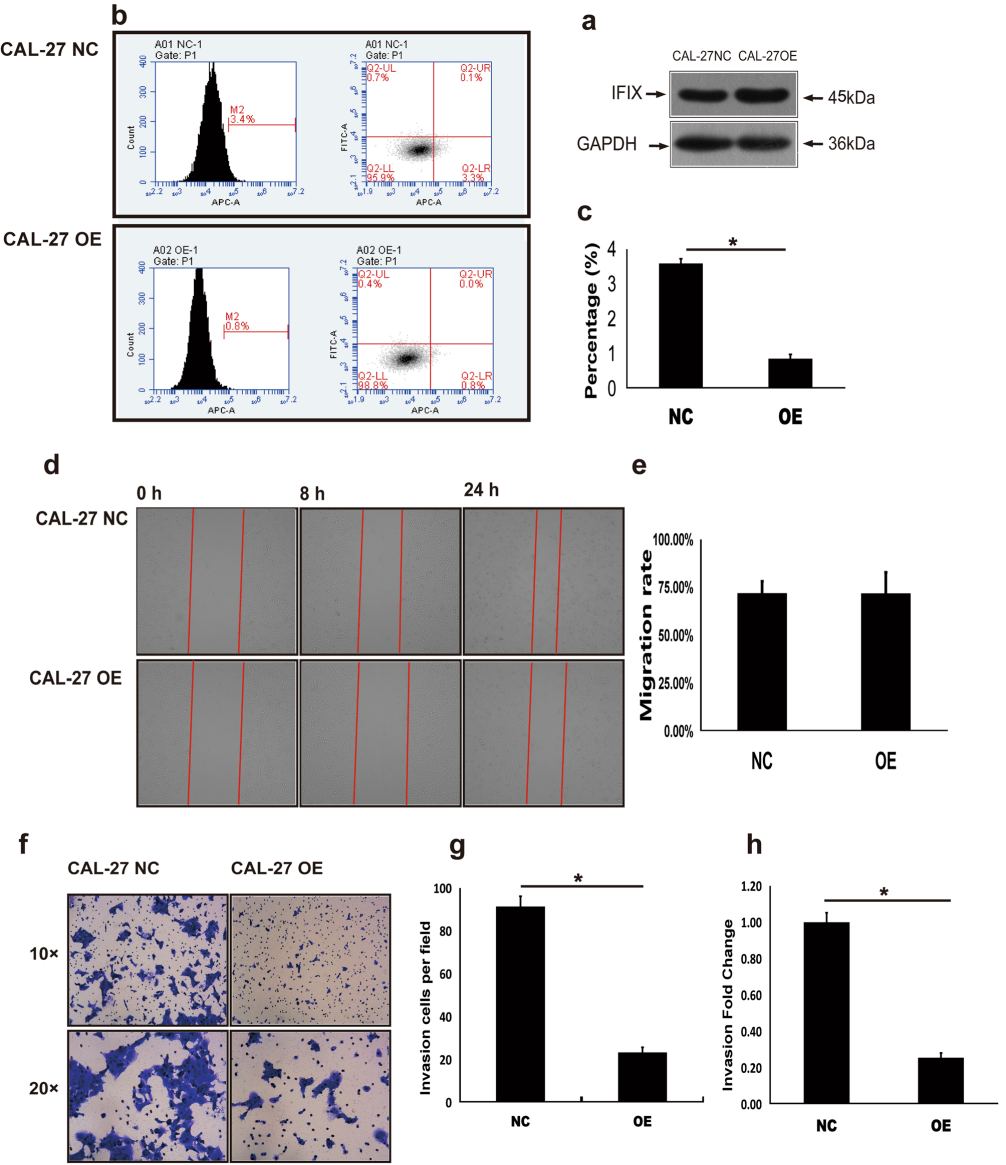
Overexpression of IFIX induces OSCC apoptosis and suppresses cell invasion. (**a**) IFIX expression in CAL-27-NC and CAL-27-OE cells by western bloting and the blots were cut prior to hybridisation with antibodies during blotting. (**b**) Analysis of apoptosis induced in OSCC by overexpression of IFIX. Representative flow plots are shown. Apoptosis was analyzed using a FACS Calibur flow cytometer (BD Biosciences, San Jose, CA, USA) with a FITC Annexin V Apoptosis Detection kit (BD Biosciences). (**c**) Apoptosis rate measured by annexin V staining and determined by flow cytometry. The apoptosis percentage is presented as the mean ± SEM (n = 3); *P < 0.05 vs. control. All data were analyzed using FCS Express software (version 3.0; De Novo Software, Pasadena, CA, USA). (**d**) The wound healing assay indicates that overexpression of IFIX does not inhibit CAL-27 cell migration (magnification 100 ×). Representative pictures are given. (**e**) Data are presented as the mean ± SD (n = 3); *P < 0.05 vs. control. (**f**) Transwell assay revealing that IFIX overexpression significantly inhibited CAL-27 cell invasion. Samples from three independent assays and representative pictures are shown (magnification 100 ×  or 200 ×). (**g**,**h**) Graphs indicate invading cells in fields of view and an invasion fold change .

Because tumor-suppressor activity of IFIXa1 has been found in breast cancer^10,12^, we next attempted to determine if this tumor-suppressive effect extended to OSCC. Lentivirus expression vectors were transfected into CAL-27 oral tongue cancer cells (CAL-27-NC, control: no IFIX overexpression), and stably transfected IFIX-overexpressing cells (CAL-27-OE) were selected for experiments. Expression of IFIX protein in CAL-27-NC and CAL-27-OE cells was confirmed by western blotting (Fig. 2a). Next, apoptosis, cell migration, and cell invasiveness were evaluated. In a fluorescence-activated cell sorting (FACS) apoptosis assay, CAL-27-OE cells showed a higher percentage of apoptotic cells than CAL-27-IFIX-NC (Fig. 2b,c). However, in a wound-healing assay, migration into the scratch area by CAL-27-OE cells was not significantly reduced compared to CAL-27-NC (Fig. 2d,e). In the invasion assay, the percentage of invading CAL-27-OE cells was significantly lower than CAL-27-IFIX-NC (Fig. 2f–h). Therefore, overexpression of IFIX significantly reduced the malignant properties of CAL-27 oral tongue cancer cells.

### IFIX suppresses malignant transformation of somatic cells

**Figure 3:**
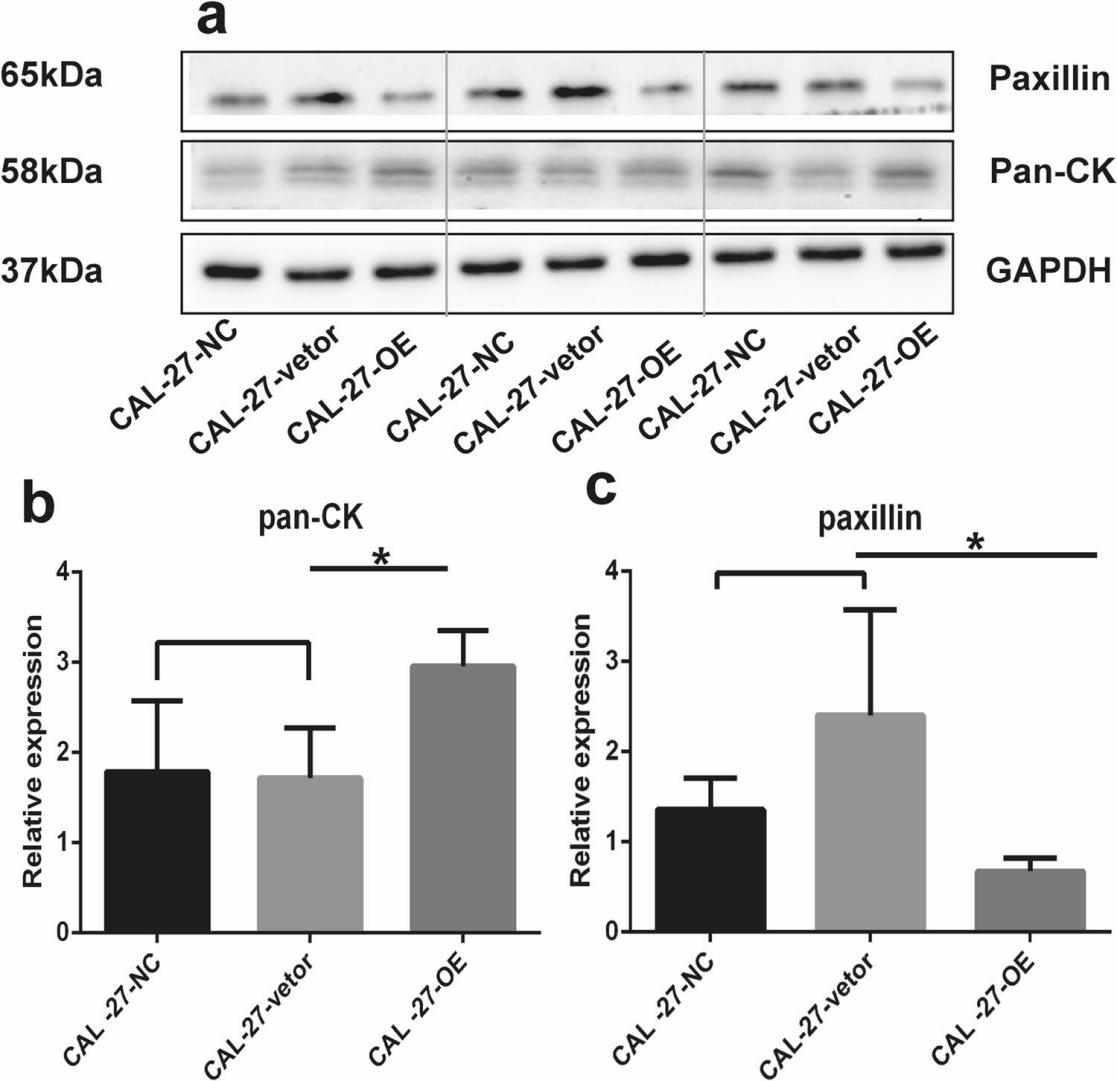
IFIX suppresses malignant transformation of somatic cells. (**a**) Analysis of various cytokeratins (with a pan-CK antibody) and paxillin by western blot in lysates of IFIX-overexpressing (CAL-27-OE), empty lentiviral vector (CAL-27-vector), and normal control CAL-27 cells (CAL-27-NC). Gels and blots were presented by three groups at a time and the blots were cut prior to hybridisation with antibodies during blotting. (**b**,**c**) The data were replicated twice in one test and presented as the mean ± SD; *P < 0.05.

Because IFIX is mainly expressed in the normal oral mucosa, at precancerous stages, or in well-differentiated OSCC and inhibits the oncogenicity of oral tongue cancer cells, we sought to investigate whether IFIX plays an essential role in somatic cell malignant transformation. From our initial data, we deduced that OSCC proliferation and invasion were inhibited by IFIX expression. To determine the underlying molecular mechanism, western blotting analysis was performed on cytoskeletal proteins, including various cytokeratins (by means of a pan-CK antibody) and paxillin. Cytokeratins are keratin-containing intermediate filaments in the intracytoplasmic cytoskeleton of epithelial tissues^14^. Paxillin is involved in epithelial morphogenesis, implying its participation in endothelial cell barrier dysfunction, which leads to abnormal cell movement and migration^15^. Our results revealed that various cytokeratins were upregulated (Fig. 3a,b) by IFIX overexpression, whereas the expression of paxillin was significantly lower in CAL-27-OE cells (Fig. 3c). Thus, IFIX can maintain and stabilize the intracytoplasmic cytoskeleton of epithelial cells.

### IFIX does not suppress the establishment of CSCs in cell culture based on the levels of CSC protein markers

**Figure 4:**
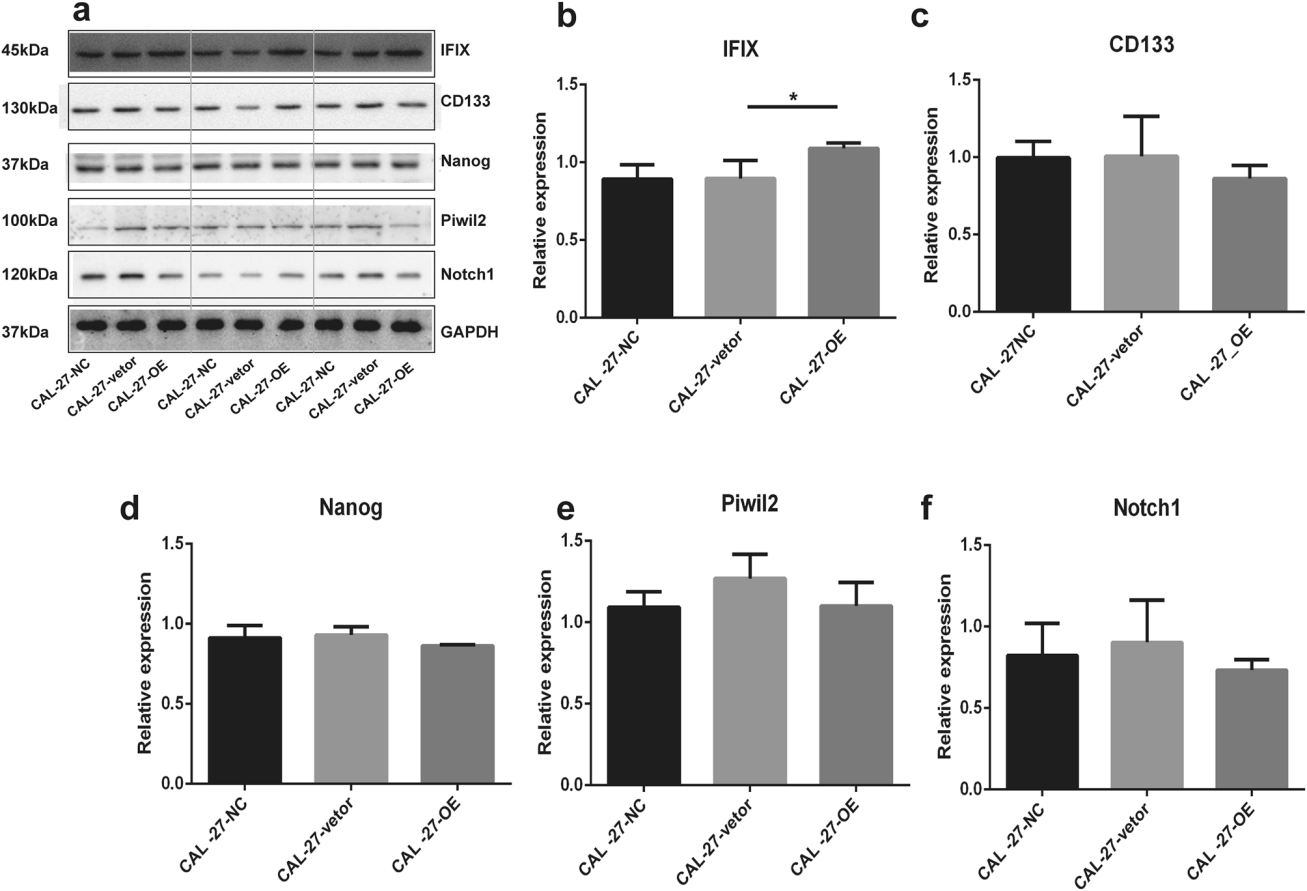
IFIX does not limit the establishment of CSCs. (**a**) Cell lysates were analyzed by western blotting for CD133, NANOG, PIWIL2, and NOTCH1 as general CSC markers in CAL-27 cells infected with the lentivirus overexpressing IFIX (CAL-27-OE), CAL-27 cells infected with the empty lentiviral vector (CAL-27-vector), and CAL-27 normal control cancer cells (CAL-27-NC) Gels and blots were presented by three groups at a time and the blots were cut prior to hybridisation with antibodies during blotting. (**b**–**f**) The data were replicated twice in one test and presented as the mean ± SD; *P < 0.05.

CSCs, which are believed to be tumor-initiating cells, exhibit the ability to initiate and propagate tumors and have a characteristic gene expression signature of embryonic stem cells^16^. We found that IFIX can maintain and stabilize the intracytoplasmic cytoskeleton of epithelial cells. Therefore, we made an assumption that IFIX may also limit the ability of somatic cells to be reprogrammed into tumor-initiating cells. Human normal oral mucosa cells (RKQNMSP_2), CAL-27-OE, and CAL-27-NC, were therefore subjected to RNA sequencing (RNA-seq). A heat map of gene expression data is presented in Supplementary Data 1. A total of 248 differentially expressed genes or new RNA fragments were identified in the three groups (Supplementary Data 2). Unfortunately, this list did not include general CSC markers, such as SOX2, PIWIL2, NOTCH1, NANOG, OCT-4, ALDH1, and CD44^17–19^. We next performed KEGG analysis on the set of 248 genes/RNAs, with a focus on typical CSC signals (with significance at *P* < 0.05) and identified 22 signaling pathways mainly associated with an innate immune reaction and cellular metabolic activity and not directly related to CSC behavior (Supplementary Data 3)^20^. We tried to validate NANOG, NOTCH1, CD133 and PIWIL2 by western blotting but we observed that variation in IFIX expression did not correlate with the expression level of these CSC protein markers (Fig. 4a–f).

## Discussion

IFIX, the most recently identified DNA sensor in the PYHIN family, plays a sentinel role in carcinogenesis, although this phenomenon was illustrated in viral infection studies^6,12,21,22^. Previous work has demonstrated that IFIX functions as a tumor suppressor in breast cancer pathogenesis^11,13,23^. Our data here also indicates that IFIX can inhibit the progression of OSCC. The oral mucosa progresses histopathologically from dysplasia to invasive cancer through a multistep cascade process. IFIX is mainly expressed in cell nuclei at histopathological stages from oral normal mucosa to hyperplasia, and IFIX may also localized to the cytoplasm upon dysplasia with abundant immune-cell invasion of the submucosa and in invasive carcinoma. Although IFIX is recognized as a nuclear DNA sensor in the PYHIN family, it has the ability to cross the nuclear membrane and bind to viral DNA in the cyto plasm through its HIN-200 domain, which initiates an innate immune response via induction of interferon β during a viral infection^21^. We cannot rule out that DNA sensors acting on fragments from the DNA damage response share a similar mechanism in carcinogenesis. In airway epithelial cells, Massa et al. have found that PYHIN1, namely IFIX, can regulate proinflammatory-cytokine induction rather than DNA sensing for innate immunity^24^. In addition, we found that the level of IFIX expression varies and depends on the differentiation status of oral carcinoma to some degree and is consistent with the role of a tumor suppressor.

To identify the mechanism of action of IFIX in carcinogenesis suppression, we established an IFIX-overexpressing cell line (CAL-27-OE) and observed that IFIX could promote cancer apoptosis and limit tumor cell invasion, and that this process is closely associated with cytoskeletal proteins in cancer cells. Therefore, we chose to validate these observations with various cytokeratins and paxillin. The biological functions of cytokeratins support relatively static phenomena in terms of supporting the shape of the nucleus and providing tensile strength to the cell^14^, whereas paxillin serves as a platform for the recruitment of numerous regulatory and structural proteins that together control dynamic changes in cell adhesion, cytoskeletal reorganization, and gene expression that are necessary for cell migration and survival^15^. Our results revealed that IFIX can stabilize cytoskeletal proteins and limit migratory ability, which may have important implications for the inhibition of cancer progression and cell proliferation. Another research group in our team is currently investigating the role of IFIX in EMT to fully identify the IFIX-containing pathway that induces this process.

Numerous studies have indicated that the metastasis of malignant cells originates from CSCs, which possess the pronounced characteristics of stem cells^25^ and play a key role in the growth, progression, and survival of tumor cells. Stemness primarily refers to the self-renewal of cells that allows primitive cells to generate differentiated cells. Stem cells can interact with the environment, the result of which affects proliferation and growth in cells, ultimately maintaining normal tissue homeostasis. CSCs, however, usurp this function to maintain cell malignancy qualities and survival status^26,27^. Here, we provide preliminary RNA-seq data on the effect of IFIX expression in OSCC CSCs (without considering the tumor microenvironment) using a normal oral mucosal cell (RKQNMSP_2), oral cancer cell (CAL-27-NC) and IFIX-overexpressing (CAL-27-OE) cell line. Unfortunately, we did not detect significant differences in the RNA expression of CSC markers between these three cell lines. Furthermore, signaling pathways potentially related to CSC functions were not found to be enriched among the differentially expressed gene/RNA set at a chosen threshold (*P* < 0.05). Neither did differences in the expression of general CSC markers at the protein level reach statistical significance. We cannot completely rule out that IFIX has some influence on oral cancer carcinogenesis through the CSC pathway. The tumor microenvironment should be key for further research in this area because of the initiation of a remarkable immune reaction signaling pathway according to KEGG analysis of differential gene expression in CAL-27-OE cells. Second, members of the WNT family, such as WNT3A, WN4, and NKD2, which are intimately linked with stem cell characteristics, were found among the significantly differentially expressed mRNAs. These phenomena may not fully support a pure tumor suppressor role of IFIX, and IFIX function may be complicated in the tumor microenvironment when considering other cofactors. Indeed, Nakamura et al. found that overexpression of AIM2, another member of the PYHIN family, contributes to tumor progression via EMT in OSCC^9^. In contrast, Man et al. reported that AIM2-deficient mice develop a larger volume colon cancer tumors in a model of carcinogenic agent-induced colitis-associated tumorigenesis^10^.

In summary, this study indicates that IFIX acts as a tumor suppressor and suppresses OSCC through stabilization of the cytoskeleton, through components such as cytokeratin, and by downregulating paxillin. The latter serves as a scaffolding for the recruitment of numerous regulatory and structural proteins, and the biological function of this process can control the dynamic changes in cell adhesion, cytoskeletal interaction, and gene expression that are essential for cell migration and survival. These results indicate that the inhibitory mechanism is directly associated with EMT. Regarding other possible mechanisms contributing to metastasis, CSCs were not affected directly by IFIX using in vitro cell culture studies by this work.

## Materials and methods

### Patients and tissue specimens

The specimens included seven normal oral mucosa tissue samples, eight oral hyperplasia tissue samples, 11 oral dyaplasia tissue samples adjacent to the tumor tissue, 15 samples of well-differentiated OSCC, and seven samples of moderate and poorly differentiated oral cancer. Oral neoplasm specimens were obtained from patients who underwent surgery. None of the patients received any tumor-specific therapy before surgical excision.

### Ethical statement

The procedures were approved by the First Affiliated Hospitals of Harbin Medical University Institutional Research Board and were consistent with the Declaration of Helsinki. Informed consent was obtained from all the patients.

### Tissue processing, immunohistochemistry, and evaluation of immunoreactivity

We followed previously reported protocols^17^. The polyclonal anti-IFIX antibody (1:50) used here was purchased from ThermoFisher Scientific (catalog no. PA5-25293, Waltham, MA, USA). Immunoreactivity was semiquantitatively evaluated based on staining intensity and distribution using an immunoreactivity score as follows: (intensity score) × (proportion score). The intensity score was defined as follows: 0, negative; 1, weak; 2, moderate; or 3, strong. The proportion score was defined as follows: 0, negative; 1, < 10%; 2, 11%–50%; 3, 51%–80%; and 4, > 80% positive cells. The total score ranges from 0 to 12. Stained lesion tissues were scored by two researchers who were blinded to the clinical data^28^.

### Cell culture and infection with overexpression lentivirus

Human oral tumor cancer cell line CAL-27 and human oral epithelial cell line RKQNMSP_2 were purchased from the American Type Culture Collection (ATCC; Manassas, VA, USA) and Shangcheng Beina Chuanglian Biotechnology Co. Ltd., respectively, and cultured in Dulbecco’s Modified Eagle Medium (DMEM) or RPMI-1640 with 10% fetal bovine serum. To generate cell lines overexpressing IFIX, CAL-27 cells were transfected with lentiviral LV-PYHIN1(43933-2)-EGFP (Genechem, Shanghai, China) or empty vector lentiviruses expressing GFP (Genechem). Cells were cultured in 6-well plates until 60% confluence and infected with lentivirus particles in the presence of 10 g/mL polybrene at an MOI of 10. The cells were cultured for at least 96 h before further experiments were performed.

### Apoptosis assays

Apoptosis was analyzed using a FACS Calibur flow cytometer (BD Biosciences, San Jose, CA, USA) with a FITC Annexin V Apoptosis Detection kit (BD Biosciences). All data were analyzed using FCS Express software (version 3.0; De Novo Software, Pasadena, CA, USA). Briefly, cells were suspended in 1 × binding buffer at a concentration of 1 × 10^6^ cells/mL. The cell suspension (200 μL) was then transferred to a 1.5-ml Eppendorf tube, mixed with 10 μL APC annexin V, and incubated for 15 min at room temperature in the dark. Within 1 h, the samples were analyzed by flow cytometry, and each experiment was performed three times.

### Wounding healing assay

CAL-27-Vector (CAL-27 -IFIX vector) and CAL-27-OE (CAL-27 -IFIX over-expression) were plated in 96-well culture dishes, serum-starved for 12 to 16 h, and wounded with a 96 Wounding Repicator. The culture dishes were washed three times with 0.5% FBS to remove detached cells, and the remaining cells were grown in DMEM containing 10% FBS. After 8 h and 24 h of incubation, migration was quantified by counting the cells (Geligo) that had moved beyond the reference line.

### Invasion assay

In vitro invasion assay experiments were conducted using Corning BioCoat Matrigel Invasion Chambers (Corning, NY, USA). Briefly, DMEM containing 30% FBS was placed in the lower wells, and CAL-27 cells were suspended at a final concentration of 1 × 10^5^ cells/mL in serum-free DMEM in the upper wells. The plates were incubated at 37 °C in 5% CO_2_ for 40 h. After incubation, non-migrating cells were removed from the upper surface of the filter using a cotton swab. Two-to-three drops of Giemsa staining fluid were added to the filters for 3–5 min. Invasion was quantified using an optical microscope to count the stained cells that had migrated to the lower side of the filter. The stained cells were counted as the mean number of cells per nine random fields for each assay.

### Western blot analysis

Protein concentrations were determined using a Bicinchoninic Acid Protein Assay Kit (ThermoFisher Scientific). Equal amounts of protein were solubilized in Laemmli buffer (62.5 mM Tris–HCl, pH 6.8, 10% glycerol, 2% SDS, 5% β-mercaptoethanol, and 0.00625% bromophenol blue), boiled for 5 min, separated by SDS-PAGE, and transferred onto nitrocellulose membranes. The membranes were probed with the following primary antibodies: anti-pan-cytokeratin (BM0030, Boster Bio, Pleasanton, CA, USA), anti-paxillin (22172-1-AP Proteintech Group, Chicago, IL, USA), anti-IFIX (NBP1-79436,Bio-Techne), anti-Piwil2 (ab85084, Abcam, Inc., Cambridge, UK), anti-Nanog (bs-10408R, Biotechnology, Beijing, China), anti-Notch1 (bs-1335R, Biotechnology), anti-CD133 (bs-4770R, Biotechnology), and anti-GAPDH (G8795, MilliporeSigma, Burlington, MA, USA) in TBS/Tween-20 containing 5% nonfat milk at 4 °C overnight. The membranes were incubated with the appropriate secondary antibodies for 1 h at room temperature. Immunoreactive bands were visualized using enhanced chemiluminescence (GE Healthcare, Chicago, IL, USA). The blots were cut prior to hybridisation with antibodies during blotting. Each blot is representative of at least three similar independent experiments and analyzed using the Gel-Pro Analyzer 4.0 program for Windows (Media Cybernetics, Inc., Rockville, MD, USA).

### RNA sequencing and read mapping

Cell samples were sent to Shanghai Majorbio Bio-pharm Technology Co., Ltd. (Shanghai, China). Total RNA was extracted from the tissue using TRIzol Reagent according to the manufacturer’s instructions (Invitrogen, Carlsbad, CA, USA)^29,30^ and genomic DNA was removed using DNase I (TaKara, Kusatsu, Japan). RNA quality was determined using a 2100 Bioanalyzer (Agilent, Santa Clara, CA, USA) and quantified with a ND-2000 (NanoDrop Technologies). Only high-quality RNA samples (OD260/280 = 1.8 − 2.2, OD260/230 ≥ 2.0, RIN ≥ 6.5, 28S:18S ≥ 1.0, > 2 μg) were used to construct the sequencing library. The RNA-seq transcriptome library was prepared using the TruSeq RNA sample preparation Kit from Illumina (San Diego, CA, USA) using 1 μg of total RNA. Briefly, messenger RNA was isolated according to the polyA selection method using oligo (dT) beads and then fragmented using fragmentation buffer. Second, double-stranded cDNA was synthesized using a SuperScript double-stranded cDNA synthesis kit (Invitrogen) with random hexamer primers (Illumina). Then the synthesized cDNA was subjected to end-repair, phosphorylation, and ‘A’ base addition according to Illumina’s library construction protocol. Libraries were selected for cDNA target fragments of 200–300 bp on 2% Low Range Ultra Agarose, followed by PCR amplification using Phusion DNA polymerase (New England Biolabs, Ipswich, MA, USA) for 15 PCR cycles. After quantification by TBS380, the paired-end RNA-seq sequencing library was sequenced using the Illumina HiSeq xten/NovaSeq 6000 sequencer (2 × 150 bp read length).

Raw paired-end reads were trimmed and quality controlled by SeqPrep (https://github.com/jstjohn/SeqPrep) and Sickle (https://github.com/najoshi/sickle) with default parameters. Then, clean reads were separately aligned to the reference genome in orientation mode using TopHat (http://tophat.cbcb.umd.edu/, version2.0.0) software^31^. The mapping criteria of Bowie were as follows: sequencing reads were uniquely matched to the genome, allowing up to two mismatches, without insertions or deletions. Then, the region of the gene was expanded following the depths of the sites, and the operon was obtained. In addition, the whole genome was split into multiple 15 kbp windows that share 5 kbp. New transcribed regions were defined as more than two consecutive windows without overlapping regions of the gene, where at least two reads were mapped per window in the same orientation.

### Bioinformatics and statistical analysis

To identify differentially expressed genes (DEGs), the expression level of each transcript was calculated according to the fragments per kilobase of exon per million mapped reads (FRKM) method. RSEM (http://deweylab.biostat.wisc.edu/rsem/)^32^ was used to quantify the gene abundance. The R statistical package software EdgeR (Empirical analysis of Digital Gene Expression in R, http://www.bioconductor.org/packages/2.12/bioc/html/edgeR.html)^33^ was used for differential expression analysis. In addition, functional enrichment analysis, including GO and KEGG, was performed to identify which DEGs were significantly enriched in GO terms and metabolic pathways at Bonferroni-corrected *P* value ≤ 0.05, compared with the whole-transcriptome background. GO functional enrichment and KEGG pathway analyses were carried out using Goatools (https://github.com/tanghaibao/Goatools) and KOBAS (http://kobas.cbi.pku.edu.cn/home.do)^34^. Data are expressed as the mean ± SEM. The data presented were derived from at least three independent in vitro experiments. To reduce baseline variability between independent experiments, quantitative analysis of immunoblots and mRNA expression were normalized. Data were normalized to the fold change over the mean of the control. Two-group comparisons were made using a 2-tailed Student’s *t*-test; three or more different groups were evaluated by one-way ANOVA followed by Bonferroni’s post hoc comparisons. Statistical significance was set at *P* < 0.05. Statistical analyses of the data were performed using the GraphPad Prism 6.

## Supplementary Information

Supplementary Information.
