# Supplementary material for: Interferon-inducible protein, IFIX, has tumor-suppressive effects in oral squamous cell carcinoma
Source: Sci Rep. 2021 Oct 1;11:19593. doi: 10.1038/s41598-021-99157-4 (PMC8486792; doi:10.1038/s41598-021-99157-4)
Supplement: Supplementary file 1 — Supplementary Information. [file 41598_2021_99157_MOESM1_ESM.pdf]

Interferon-inducible protein, IFIX, has tumor-suppressive effects in oral squamous cell carcinoma

Shan Wang, PhD , Fang Li, M.M , Haixia Fan, PhD

Figure legend

> For Figure.2 supplementary information, the original western blotting pictures including IFIX (also named PYHIN1)and GAPDH were shown. **1.NC** represented CAL-27-NC; **2.OE** represented CAL-27-OE.

> For Figure.3 supplementary information, the original western bloting pictures in the Fig.3a including Paxillin, pan-CK and GAPDH were shown.

>For Figure.4 supplementary information. The original western bloting pictures in the Fig.4a including IFIX,CD133, Nanog, Piwil2,Notch1 and GAPDH were shown.

> For Figure.4 supplementary information. A fuller-length original, un-processed blot of PHYIN1(IFIX) were presented, in which the left-most column were the marker strips, and the next were CAL-27-vector and CAL-27-OE respectively. Residual four blots respectively duplicated in CAL-27-vector and CAL-27-OE twice.

> Supplementary data 1 showed that a heat map of gene expression data obtained from RKQNMSP_2, CAL-27-OE, and CAL-27-NC cells.

> Supplementary data 2 showed that A total of 248 specifically expressed genes or new RNA fragments were identified in the three groups from RKQNMSP_2, CAL-27-OE, and CAL-27-NC cells.

> Supplementary data 3 showed the set of 248 genes/RNAs for enrichment with potential CSC signals by KEGG analysis

For Figure.2 supplementary information, the original western blotting pictures including IFIX (also named PYHIN1)and GAPDH were shown. 1.NC represented CAL-27-NC; 2.OE represented CAL-27-OE.

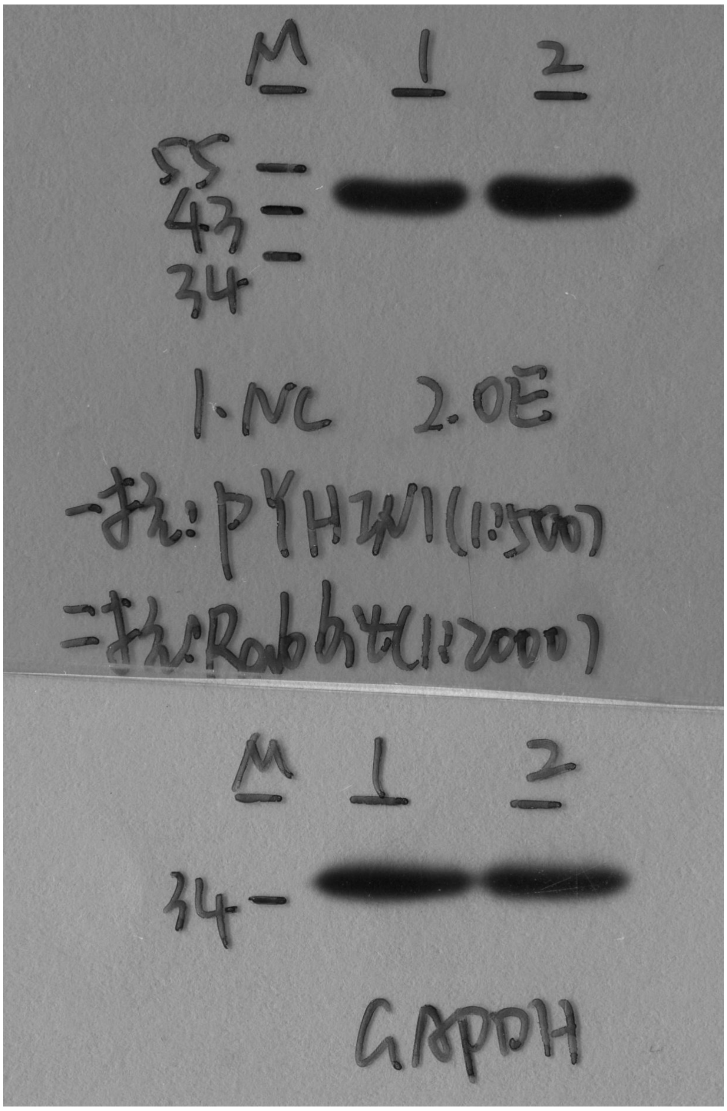

The original western bloting pictures in Fig.3a

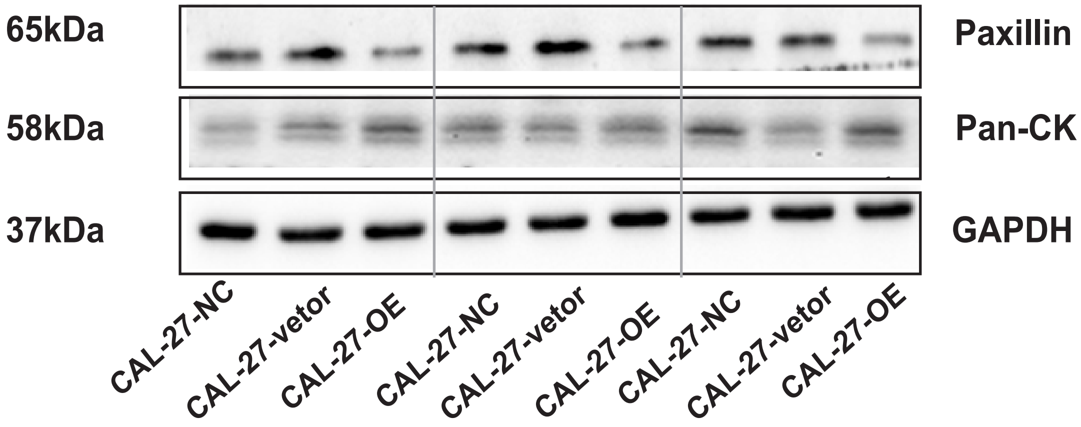

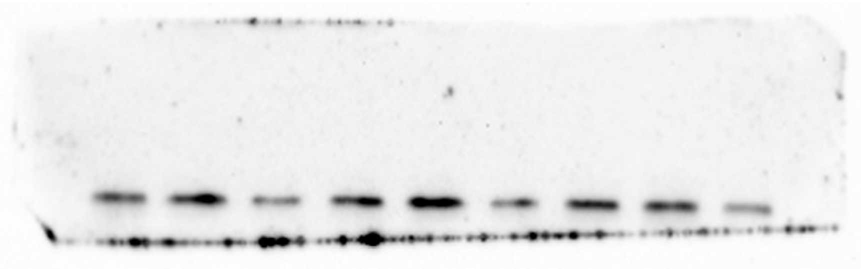

Paxillin-original

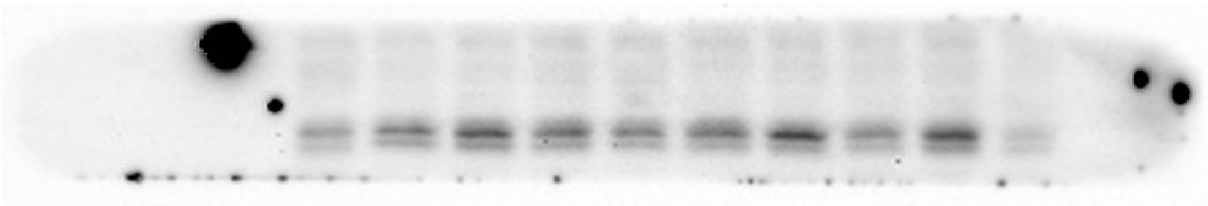

Pan-CK-original

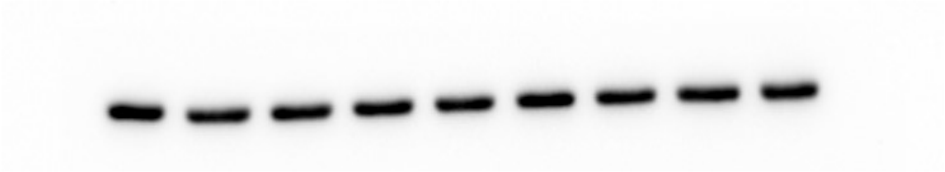

GAPDH -original

The original western bloting pictures in the Fig.4a including IFIX,CD133 and Nanog

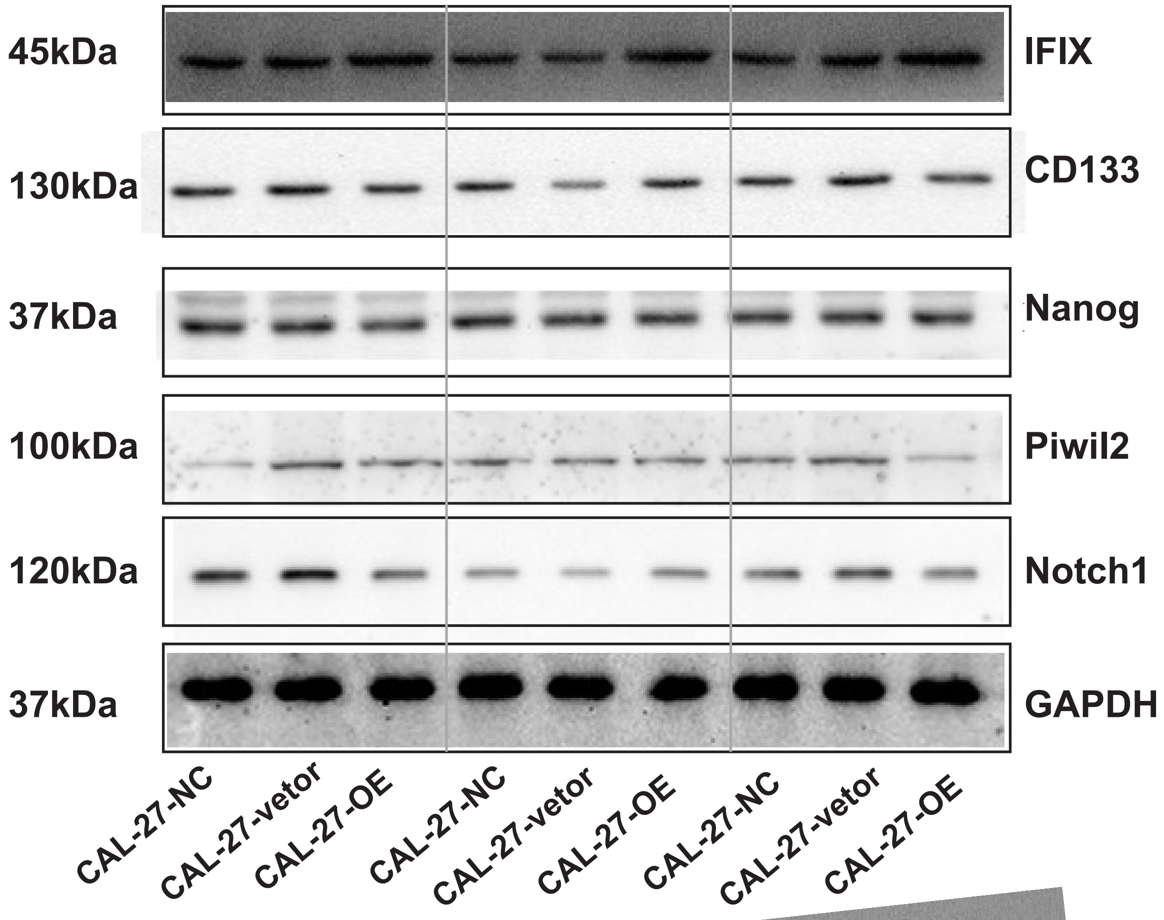

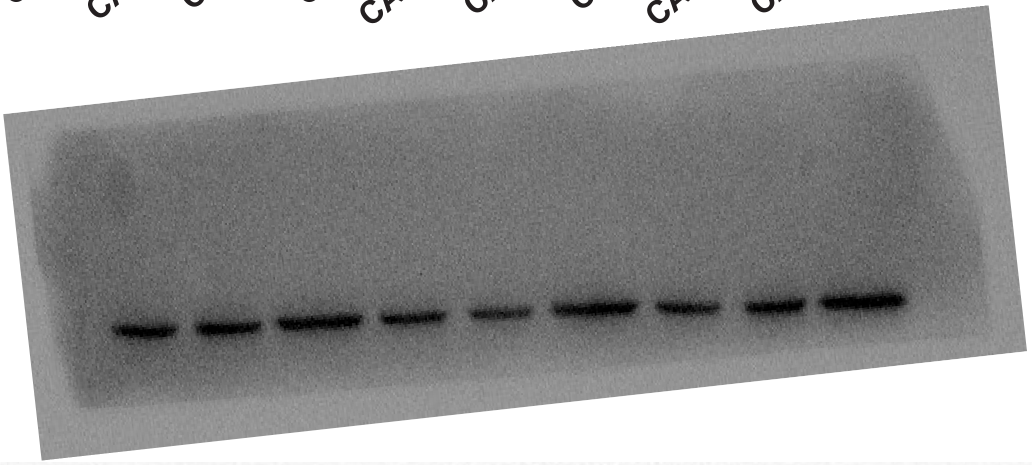

IFIX-original

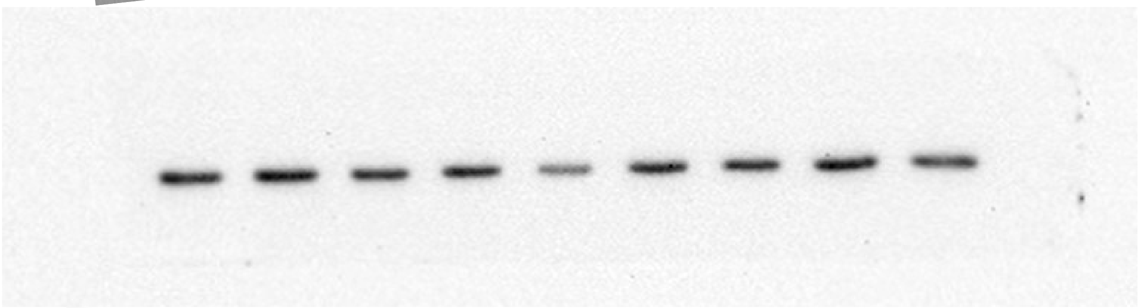

CD133-original

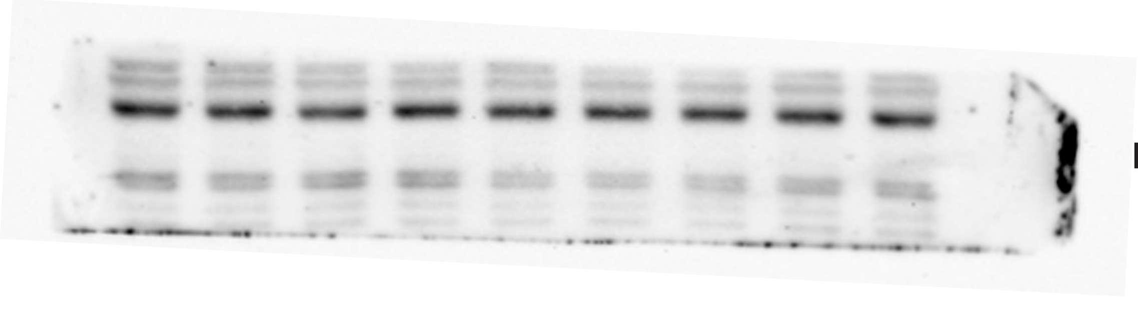

Nanog-original

The original western bloting pictures in figur.4a including Piwil2, Notch1 and GAPDH.

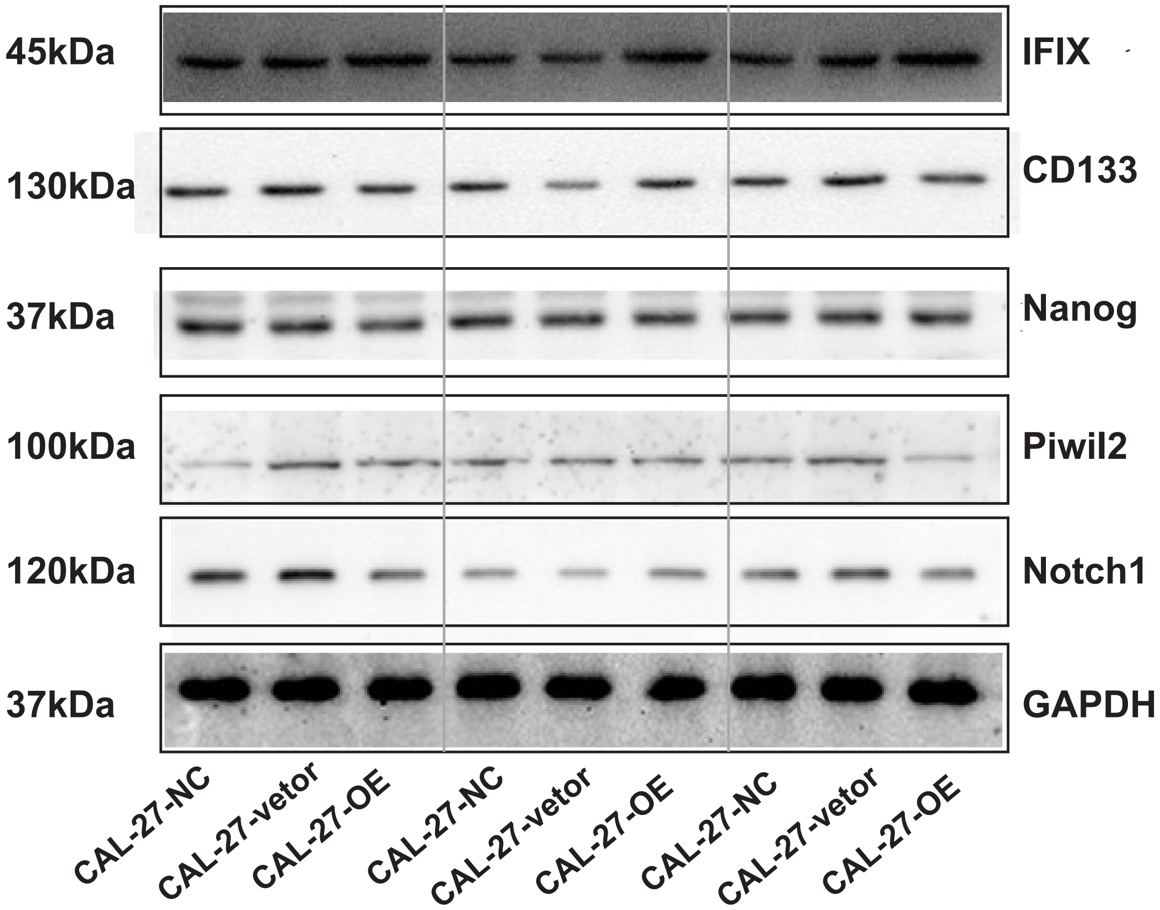

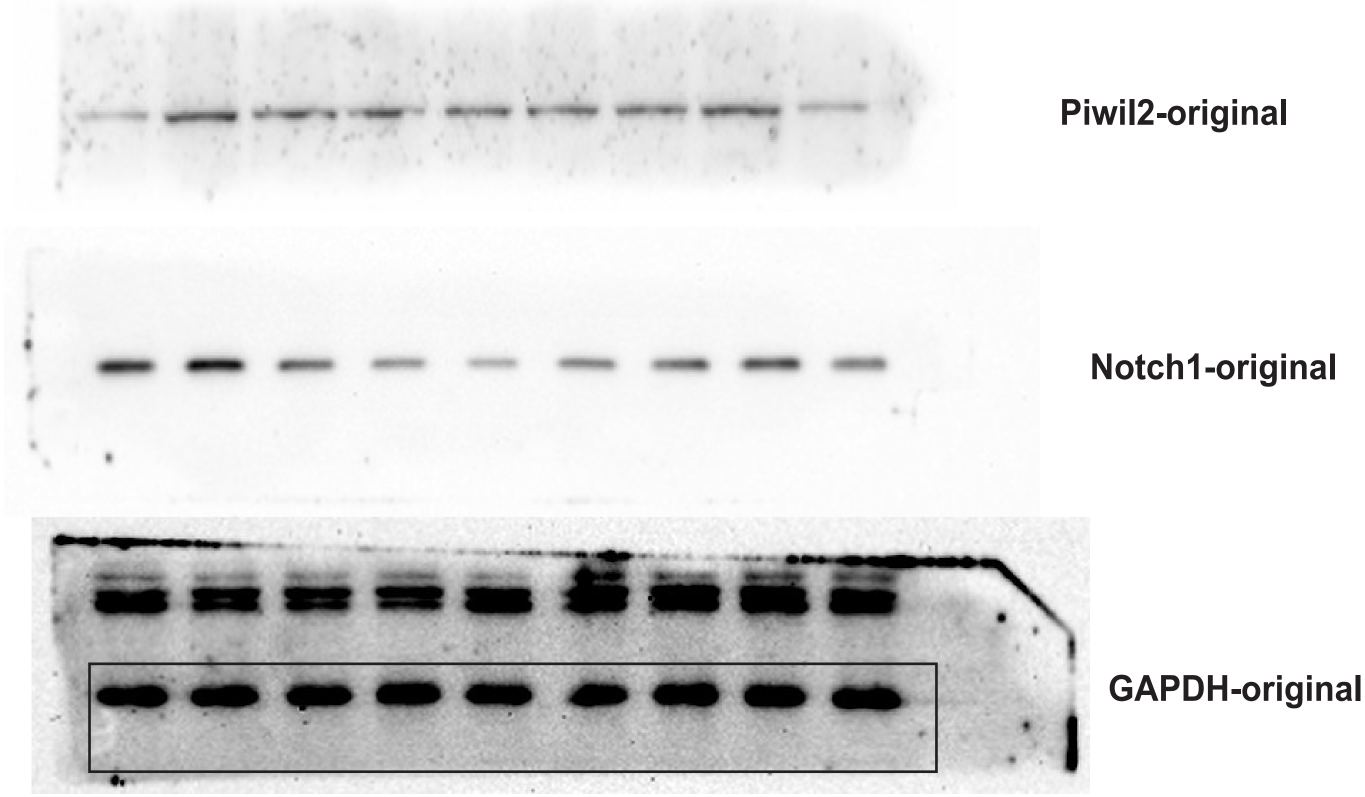

The full original un-processed western bloting pictures in figur.4a IFIX and GAPDH.

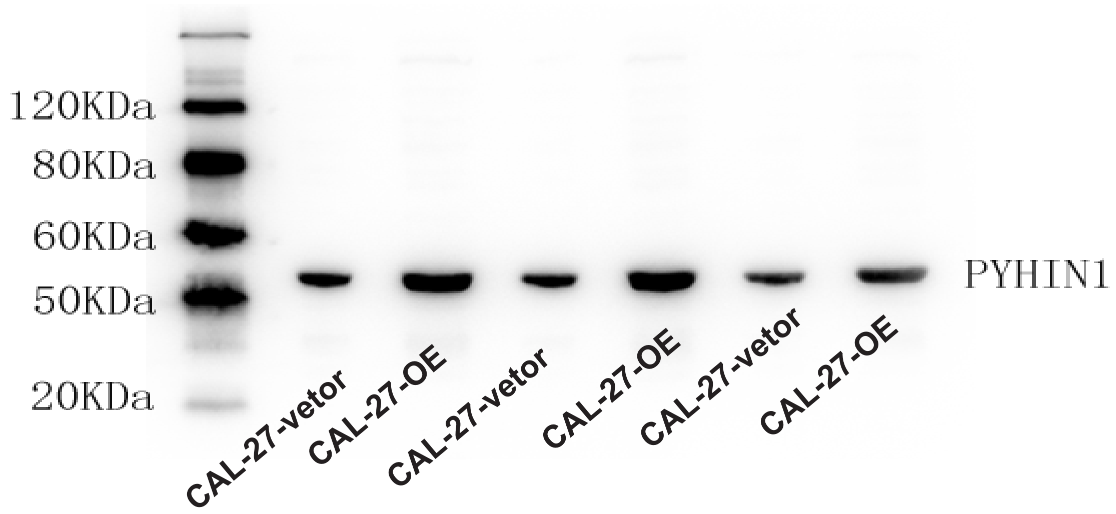

IFIX (PYHIN1)

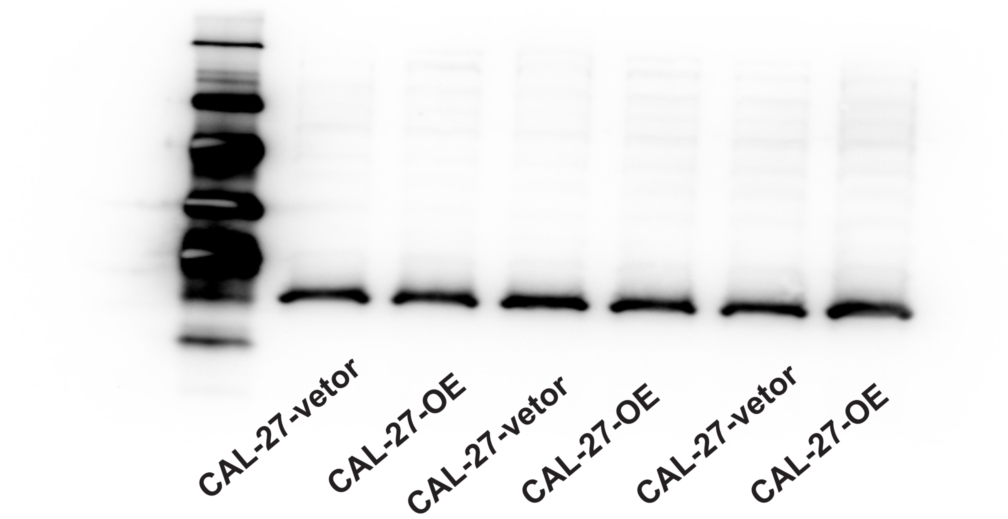

GAPDH

Interferon-inducible protein, IFIX, has tumor-suppressive effects in oral squamous cell carcinoma

Shan Wang, PhD , Fang Li, M.M , Haixia Fan, PhD

Supplementary data 1 showed that a heat map of gene expression data obtained from RKQNMSP_2, CAL-27-OE, and CAL-27-NC cells

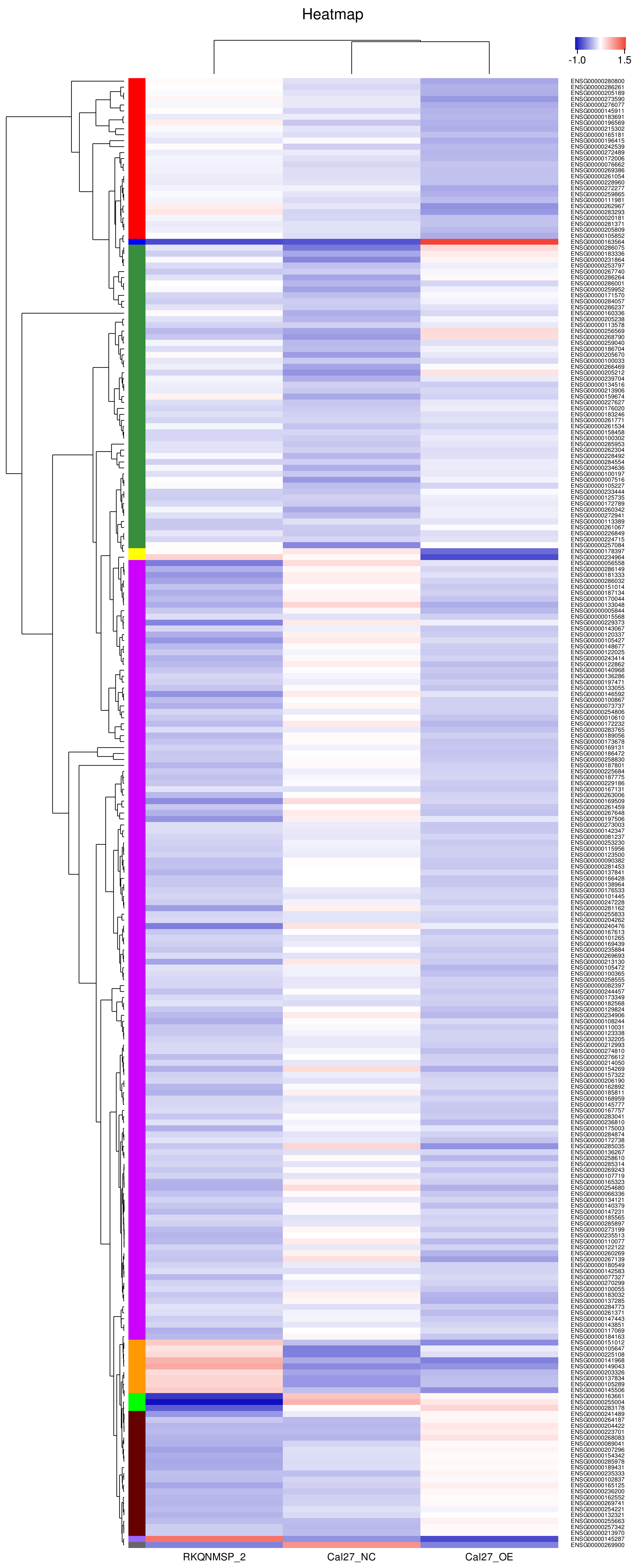
Heatmap
-1.0
1.5
RKQNMSP_2
Cal27_NC
Cal27_OE

Interferon-inducible protein, IFIX, has tumor-suppressive effects in oral squamous cell carcinoma

Shan Wang, PhD , Fang Li, M.M , Haixia Fan, PhD

Supplementary data 2 showed that A total of 248 specifically expressed genes or new RNA fragments were identified in the three groups from RKQNMSP_2, CAL-27-OE, and CAL-27-NC cells.

Gene_id	Gene name
ENSG00000280800	FP671120.4
ENSG00000163564	PYHIN1
ENSG00000286075	AC009412.1
ENSG00000178397	FAM220A
ENSG00000056558	TRAF1
ENSG00000286149	FP236383.9
ENSG00000151012	SLC7A11
ENSG00000183336	BOLA2
ENSG00000105647	PIK3R2
ENSG00000163661	PTX3
ENSG00000181333	HEPHL1
ENSG00000255004	AL133330.2
ENSG00000286032	FP236383.4
ENSG00000241489	AC244197.3
ENSG00000151014	NOCT
ENSG00000187134	AKR1C1
ENSG00000170044	ZPLD1
ENSG00000264187	AC055811.2
ENSG00000133048	CHI3L1
ENSG00000005844	ITGAL
ENSG00000231864	AL807752.3
ENSG00000015568	RGPD5
ENSG00000253797	UTP14C
ENSG00000286261	AC022137.3
ENSG00000229373	LINC00452
ENSG00000267740	AC024592.3
ENSG00000204422	AL662899.1
ENSG00000205189	ZBTB10
ENSG00000286264	AP001453.5
ENSG00000273590	SMIM11B
ENSG00000143067	ZNF697
ENSG00000276077	CU633904.1
ENSG00000225108	ZBTB45P1
ENSG00000120337	TNFSF18
ENSG00000105427	CNFN
ENSG00000148677	ANKRD1
ENSG00000223701	RAET1E-AS1
ENSG00000122025	FLT3
ENSG00000243414	TICAM2
ENSG00000286001	AC021087.5
ENSG00000259952	AC009133.2
ENSG00000140968	IRF8
ENSG00000122862	SRGN
ENSG00000268083	AC008982.1
ENSG00000136286	MYO1G
ENSG00000197471	SPN

ENSG00000171570	RAB4B-EGLN2
ENSG00000133055	MYBPH
ENSG00000146592	CREB5
ENSG00000100867	DHRS2
ENSG00000073737	DHRS9
ENSG00000254806	SYS1-DBNDD2
ENSG00000172232	AZU1
ENSG00000010610	CD4
ENSG00000283765	AC131160.1
ENSG00000145911	N4BP3
ENSG00000189056	RELN
ENSG00000089041	P2RX7
ENSG00000173678	SPDYE2B
ENSG00000284057	AP001273.2
ENSG00000286237	ARMCX5-GPRASP2
ENSG00000169131	ZNF354A
ENSG00000186472	PCLO
ENSG00000160336	ZNF761
ENSG00000258830	AC137834.1
ENSG00000141968	VAV1
ENSG00000187801	ZFP69B
ENSG00000225684	FAM225B
ENSG00000187775	DNAH17
ENSG00000183691	NOG
ENSG00000149043	SYT8
ENSG00000205238	SPDYE2
ENSG00000207296	RNU6-140P
ENSG00000229186	ADAM1A
ENSG00000196569	LAMA2
ENSG00000113578	FGF1
ENSG00000167131	CCDC103
ENSG00000256569	AC069234.3
ENSG00000154342	WNT3A
ENSG00000203326	ZNF525
ENSG00000263006	ROCK1P1
ENSG00000268790	AC008764.4
ENSG00000259040	BLOC1S5-TXNDC5
ENSG00000186704	DTX2P1
ENSG00000169509	CRCT1
ENSG00000137834	SMAD6
ENSG00000261459	AC002310.5
ENSG00000285978	AC113348.2
ENSG00000267648	AC060766.5
ENSG00000197506	SLC28A3
ENSG00000189431	RASSF10
ENSG00000273003	ARL2-SNX15
ENSG00000142347	MYO1F

ENSG00000253230	LINC00599
ENSG00000081237	PTPRC
ENSG00000123500	COL10A1
ENSG00000115956	PLEK
ENSG00000205670	SMIM11A
ENSG00000215302	AC127502.1
ENSG00000090382	LYZ
ENSG00000281453	TGFB2-OT1
ENSG00000235333	PVRIG2P
ENSG00000234964	FABP5P7
ENSG00000137841	PLCB2
ENSG00000166428	PLD4
ENSG00000138964	PARVG
ENSG00000176533	GNG7
ENSG00000100033	PRODH
ENSG00000101445	PPP1R16B
ENSG00000266469	AC005288.1
ENSG00000205212	CCDC144NL
ENSG00000102837	OLFM4
ENSG00000165181	SHOC1
ENSG00000247228	AC009060.1
ENSG00000105289	TJP3
ENSG00000281162	LINC01127
ENSG00000255833	TIFAB
ENSG00000196415	PRTN3
ENSG00000145287	PLAC8
ENSG00000204262	COL5A2
ENSG00000165125	TRPV6
ENSG00000240476	LINC00973
ENSG00000167613	LAIR1
ENSG00000169439	SDC2
ENSG00000101265	RASSF2
ENSG00000235884	LINC00941
ENSG00000269693	AC010422.6
ENSG00000242539	AC007620.2
ENSG00000213130	EEF1DP5
ENSG00000236200	KDM4A-AS1
ENSG00000105472	CLEC11A
ENSG00000239704	CDRT4
ENSG00000100365	NCF4
ENSG00000145506	NKD2
ENSG00000258555	SPECC1L-ADORA2A
ENSG00000134516	DOCK2
ENSG00000082397	EPB41L3
ENSG00000244457	ENO1P1
ENSG00000162552	WNT4
ENSG00000173349	SFT2D3

ENSG00000272489	AL132656.3
ENSG00000182568	SATB1
ENSG00000172006	ZNF554
ENSG00000234906	APOC2
ENSG00000129824	RPS4Y1
ENSG00000110031	LPXN
ENSG00000108244	KRT23
ENSG00000076662	ICAM3
ENSG00000123338	NCKAP1L
ENSG00000212993	POU5F1B
ENSG00000132205	EMILIN2
ENSG00000274810	NPHP3-ACAD11
ENSG00000269386	RAB11B-AS1
ENSG00000213906	LTB4R2
ENSG00000276612	FP565260.2
ENSG00000214050	FBXO16
ENSG00000159674	SPON2
ENSG00000154269	ENPP3
ENSG00000157322	CLEC18A
ENSG00000269741	AC011473.4
ENSG00000206190	ATP10A
ENSG00000162892	IL24
ENSG00000254221	PCDHGB1
ENSG00000185811	IKZF1
ENSG00000168959	GRM5
ENSG00000227627	AL080276.2
ENSG00000176020	AMIGO3
ENSG00000132321	IQCA1
ENSG00000145777	TSLP
ENSG00000261054	AC036108.2
ENSG00000167757	KLK11
ENSG00000283041	AC008038.1
ENSG00000236810	ELOA-AS1
ENSG00000228960	OR2A9P
ENSG00000183246	RIMBP3C
ENSG00000272277	AL031963.3
ENSG00000261771	DNAAF4-CCPG1
ENSG00000175003	SLC22A1
ENSG00000284874	AC000093.1
ENSG00000261534	AL596244.1
ENSG00000255663	AP002373.1
ENSG00000259865	AL390728.6
ENSG00000172738	TMEM217
ENSG00000285035	AC091057.5
ENSG00000158458	NRG2
ENSG00000136267	DGKB
ENSG00000258610	AF111169.1

ENSG00000285314	AC002470.2
ENSG00000269243	AC008894.2
ENSG00000100302	RASD2
ENSG00000107719	PALD1
ENSG00000285953	AC000120.3
ENSG00000111981	ULBP1
ENSG00000165323	FAT3
ENSG00000254680	AC079329.1
ENSG00000262967	AC005921.2
ENSG00000140379	BCL2A1
ENSG00000134121	CHL1
ENSG00000066336	SPI1
ENSG00000262304	AC027796.3
ENSG00000257342	AC025165.2
ENSG00000228492	RAB11FIP1P1
ENSG00000147231	RADX
ENSG00000185565	LSAMP
ENSG00000285897	AC004687.2
ENSG00000284554	AL022318.4
ENSG00000273199	AP000692.2
ENSG00000235513	AL035681.1
ENSG00000234636	MED14OS
ENSG00000100197	CYP2D6
ENSG00000213970	AC006122.1
ENSG00000122122	SASH3
ENSG00000110077	MS4A6A
ENSG00000007516	BAIAP3
ENSG00000260269	AC105036.3
ENSG00000267139	AC005262.2
ENSG00000180549	FUT7
ENSG00000142583	SLC2A5
ENSG00000105227	PRX
ENSG00000077327	SPAG6
ENSG00000269900	RMRP
ENSG00000283293	RN7SK
ENSG00000233444	AC013262.1
ENSG00000270299	AL121758.1
ENSG00000125735	TNFSF14
ENSG00000100055	CYTH4
ENSG00000183032	SLC25A21
ENSG00000137285	TUBB2B
ENSG00000284773	AC114490.3
ENSG00000172789	HOXC5
ENSG00000260342	AC138811.2
ENSG00000283178	BX119927.1
ENSG00000020181	ADGRA2
ENSG00000272941	AC083862.2

ENSG00000281371	INE2
ENSG00000113389	NPR3
ENSG00000261371	PECAM1
ENSG00000147443	DOK2
ENSG00000143851	PTPN7
ENSG00000261067	AC109460.3
ENSG00000226849	AL109811.1
ENSG00000205809	KLRC2
ENSG00000224715	Z82186.1
ENSG00000105852	PON3
ENSG00000117069	ST6GALNAC5
ENSG00000184163	C1QTNF12
ENSG00000257084	MIR200CHG

Gene description	FC(Cal27_OE/C	Log2FC(Cal27_OE
novel transcript, similar to YY1 associated myogenesis RNA 1 YAM1	0.405	-1.305099694
pyrin and HIN domain family member 1 [Source:HGNC Symbol;Acc:HGN(	5953.894	12.53961789
Protein FAM220A [Source:UniProtKB/Swiss-Prot;Acc:Q7Z4H9]	30.636	4.937150429
family with sequence similarity 220 member A [Source:HGNC Symbol;Ac	0.006	-7.393450302
TNF receptor associated factor 1 [Source:HGNC Symbol;Acc:HGNC:1203	0.389	-1.363912631
TEC	0.494	-1.018515481
solute carrier family 7 member 11 [Source:HGNC Symbol;Acc:HGNC:110	0.474	-1.075898974
bolA family member 2 [Source:HGNC Symbol;Acc:HGNC:29488]	3.286	1.716513085
phosphoinositide-3-kinase regulatory subunit 2 [Source:HGNC Symbol;A	12.215	3.610621284
pentraxin 3 [Source:HGNC Symbol;Acc:HGNC:9692]	0.439	-1.188411988
hephaestin like 1 [Source:HGNC Symbol;Acc:HGNC:30477]	0.44	-1.185456248
novel transcript	0.434	-1.203702704
TEC	0.249	-2.006795216
novel protein	2.305	1.205036409
nocturnin [Source:HGNC Symbol;Acc:HGNC:14254]	0.485	-1.042676055
aldo-keto reductase family 1 member C1 [Source:HGNC Symbol;Acc:HGN	0.487	-1.03807971
zona pellucida like domain containing 1 [Source:HGNC Symbol;Acc:HGN(	0.066	-3.924599639
novel transcript	12.388	3.630877612
chitinase 3 like 1 [Source:HGNC Symbol;Acc:HGNC:1932]	0.005	-7.56202956
integrin subunit alpha L [Source:HGNC Symbol;Acc:HGNC:6148]	0.005	-7.56202956
novel transcript	9.836	3.298045085
RANBP2-like and GRIP domain containing 5 [Source:HGNC Symbol;Acc:H	0.307	-1.701746021
UTP14C, small subunit processome component [Source:HGNC Symbol;A	3.518	1.814707531
novel protein	0.116	-3.110281893
long intergenic non-protein coding RNA 452 [Source:HGNC Symbol;Acc:H	0.498	-1.006844913
novel protein	3.18	1.669213185
novel transcript	127.308	6.992175545
zinc finger and BTB domain containing 10 [Source:HGNC Symbol;Acc:HG	0.434	-1.204687199
novel protein	2.374	1.247396101
small integral membrane protein 11B [Source:HGNC Symbol;Acc:HGNC:5	0.335	-1.576576479
zinc finger protein 697 [Source:HGNC Symbol;Acc:HGNC:32034]	0.486	-1.041197397
uncharacterized LOC102724951 [Source:NCBI gene;Acc:102724951]	0.242	-2.046081737
zinc finger and BTB domain containing 45 pseudogene 1 [Source:HGNC S	108.105	6.756287281
TNF superfamily member 18 [Source:HGNC Symbol;Acc:HGNC:11932]	0.236	-2.082861723
cornifelin [Source:HGNC Symbol;Acc:HGNC:30183]	0.443	-1.174258926
ankyrin repeat domain 1 [Source:HGNC Symbol;Acc:HGNC:15819]	0.409	-1.291577682
RAET1E antisense RNA 1 [Source:HGNC Symbol;Acc:HGNC:48994]	91.34	6.513182151
fms related tyrosine kinase 3 [Source:HGNC Symbol;Acc:HGNC:3765]	0.011	-6.468920155
toll like receptor adaptor molecule 2 [Source:HGNC Symbol;Acc:HGNC:2	0.264	-1.923306001
novel protein	2.141	1.098517995
novel transcript, antisense to MAZ	3.054	1.610824773
interferon regulatory factor 8 [Source:HGNC Symbol;Acc:HGNC:5358]	0.024	-5.369384482
serglycin [Source:HGNC Symbol;Acc:HGNC:9361]	0.024	-5.369384482
novel protein	83.639	6.386104018
myosin IG [Source:HGNC Symbol;Acc:HGNC:13880]	0.135	-2.883957655
sialophorin [Source:HGNC Symbol;Acc:HGNC:11249]	0.025	-5.298995154

RAB4B-EGLN2 readthrough (NMD candidate) [Source:HGNC Symbol;Acc:	2.292	1.196767182
myosin binding protein H [Source:HGNC Symbol;Acc:HGNC:7552]	0.013	-6.262469278
cAMP responsive element binding protein 5 [Source:HGNC Symbol;Acc:H	0.493	-1.020693992
dehydrogenase/reductase 2 [Source:HGNC Symbol;Acc:HGNC:18349]	0.441	-1.181080951
dehydrogenase/reductase 9 [Source:HGNC Symbol;Acc:HGNC:16888]	0.234	-2.092544277
SYS1-DBNDD2 readthrough (NMD candidate) [Source:HGNC Symbol;Acc:	0.183	-2.446638708
azurocidin 1 [Source:HGNC Symbol;Acc:HGNC:913]	0.014	-6.146992061
CD4 molecule [Source:HGNC Symbol;Acc:HGNC:1678]	0.014	-6.146992061
presenilin associated rhomboid like [Source:NCBI gene;Acc:55486]	0.014	-6.133301857
NEDD4 binding protein 3 [Source:HGNC Symbol;Acc:HGNC:29852]	0.489	-1.032033518
reelin [Source:HGNC Symbol;Acc:HGNC:9957]	0.366	-1.450998248
purinergic receptor P2X 7 [Source:HGNC Symbol;Acc:HGNC:8537]	2.712	1.439232365
speedy/RINGO cell cycle regulator family member E2B [Source:HGNC Syr	0.016	-6.008726913
novel protein, C11orf54-MED17 readthrough	65.412	6.031473613
ARMCX5-GPRASP2 readthrough [Source:NCBI gene;Acc:100528062]	65.229	6.027434333
zinc finger protein 354A [Source:HGNC Symbol;Acc:HGNC:11628]	0.429	-1.222334465
piccolo presynaptic cytomatrix protein [Source:HGNC Symbol;Acc:HGNC:	0.032	-4.977067059
zinc finger protein 761 [Source:HGNC Symbol;Acc:HGNC:23179]	2.319	1.213428869
novel transcript	0.017	-5.915352851
vav guanine nucleotide exchange factor 1 [Source:HGNC Symbol;Acc:HG	0.06	-4.0645299
ZFP69 zinc finger protein B [Source:HGNC Symbol;Acc:HGNC:28053]	0.287	-1.803037659
family with sequence similarity 225 member B [Source:HGNC Symbol;Ac	0.346	-1.53006081
dynein axonemal heavy chain 17 [Source:HGNC Symbol;Acc:HGNC:2946]	0.376	-1.410026466
noggin [Source:HGNC Symbol;Acc:HGNC:7866]	0.314	-1.668944764
synaptotagmin 8 [Source:HGNC Symbol;Acc:HGNC:19264]	2.142	1.098934783
speedy/RINGO cell cycle regulator family member E2 [Source:HGNC Sym	4.327	2.113201515
RNA, U6 small nuclear 140, pseudogene [Source:HGNC Symbol;Acc:HGN	2.476	1.307961453
ADAM metallopeptidase domain 1A (pseudogene) [Source:HGNC Symbo	0.085	-3.548793733
laminin subunit alpha 2 [Source:HGNC Symbol;Acc:HGNC:6482]	0.158	-2.661565233
fibroblast growth factor 1 [Source:HGNC Symbol;Acc:HGNC:3665]	2.057	1.040634943
coiled-coil domain containing 103 [Source:HGNC Symbol;Acc:HGNC:327(	0.236	-2.081548745
novel transcript, antisense to UNC119B	55.678	5.799036929
Wnt family member 3A [Source:HGNC Symbol;Acc:HGNC:15983]	2.252	1.171324781
zinc finger protein 525 [Source:HGNC Symbol;Acc:HGNC:29423]	28.134	4.814226089
Rho associated coiled-coil containing protein kinase 1 pseudogene 1 [Sou	0.417	-1.263544503
novel protein	9.408	3.233860916
BLOC1S5-TXNDC5 readthrough (NMD candidate) [Source:HGNC Symbol;	5.796	2.53502971
DTX2 pseudogene 1 [Source:HGNC Symbol;Acc:HGNC:42352]	51.451	5.68513844
cysteine rich C-terminal 1 [Source:HGNC Symbol;Acc:HGNC:29875]	0.24	-2.057987055
SMAD family member 6 [Source:HGNC Symbol;Acc:HGNC:6772]	2.503	1.323592419
novel transcript	0.02	-5.632418888
novel transcript	2.694	1.429513257
DTW domain containing 2 (DTWD2) pseudogene	0.085	-3.555439751
solute carrier family 28 member 3 [Source:HGNC Symbol;Acc:HGNC:164	0.498	-1.005263951
Ras association domain family member 10 [Source:HGNC Symbol;Acc:HG	2.54	1.344861036
ARL2-SNX15 readthrough (NMD candidate) [Source:HGNC Symbol;Acc:H	0.021	-5.571615609
myosin IF [Source:HGNC Symbol;Acc:HGNC:7600]	0.073	-3.784421981

long intergenic non-protein coding RNA 599 [Source:HGNC Symbol;Acc:H	0.021	-5.56202956
protein tyrosine phosphatase, receptor type C [Source:HGNC Symbol;Acc	0.021	-5.56202956
collagen type X alpha 1 chain [Source:HGNC Symbol;Acc:HGNC:2185]	0.041	-4.620923249
pleckstrin [Source:HGNC Symbol;Acc:HGNC:9070]	0.041	-4.620923249
small integral membrane protein 11A [Source:HGNC Symbol;Acc:HGNC:1	2.908	1.540082961
WAS protein homolog associated with actin, golgi membranes and micro	0.423	-1.241154025
lysozyme [Source:HGNC Symbol;Acc:HGNC:6740]	0.119	-3.076096435
TGFB2 overlapping transcript 1 [Source:HGNC Symbol;Acc:HGNC:50629]	0.052	-4.252909385
poliovirus receptor related immunoglobulin domain containing 2, pseudo	46.696	5.545239834
fatty acid binding protein 5 pseudogene 7 [Source:HGNC Symbol;Acc:HG	0.022	-5.519325109
phospholipase C beta 2 [Source:HGNC Symbol;Acc:HGNC:9055]	0.433	-1.208392605
phospholipase D family member 4 [Source:HGNC Symbol;Acc:HGNC:237	0.043	-4.546029081
parvin gamma [Source:HGNC Symbol;Acc:HGNC:14654]	0.022	-5.497489308
G protein subunit gamma 7 [Source:HGNC Symbol;Acc:HGNC:4410]	0.075	-3.731954561
proline dehydrogenase 1 [Source:HGNC Symbol;Acc:HGNC:9453]	2.367	1.243038319
protein phosphatase 1 regulatory subunit 16B [Source:HGNC Symbol;Acc	0.042	-4.56202956
novel transcript, antisense to MED1 & FBXL20	45.68	5.513503071
coiled-coil domain containing 144 family, N-terminal like [Source:HGNC S	6.42	2.682516266
olfactomedin 4 [Source:HGNC Symbol;Acc:HGNC:17190]	3.466	1.793451095
shortage in chiasmata 1 [Source:HGNC Symbol;Acc:HGNC:26535]	0.395	-1.339637139
novel transcript, antisense to PDPR & CLEC18C	0.023	-5.470842468
tight junction protein 3 [Source:HGNC Symbol;Acc:HGNC:11829]	2.047	1.033821257
long intergenic non-protein coding RNA 1127 [Source:HGNC Symbol;Acc:	0.443	-1.173464272
TIFA inhibitor [Source:HGNC Symbol;Acc:HGNC:34024]	0.043	-4.529812996
proteinase 3 [Source:HGNC Symbol;Acc:HGNC:9495]	0.023	-5.436498678
placenta specific 8 [Source:HGNC Symbol;Acc:HGNC:19254]	0.023	-5.436498678
collagen type V alpha 2 chain [Source:HGNC Symbol;Acc:HGNC:2210]	0.249	-2.006814403
transient receptor potential cation channel subfamily V member 6 [Sourc	2.604	1.380484945
long intergenic non-protein coding RNA 973 [Source:HGNC Symbol;Acc:H	0.459	-1.122117392
leukocyte associated immunoglobulin like receptor 1 [Source:HGNC Sym	0.056	-4.168851495
syndecan 2 [Source:HGNC Symbol;Acc:HGNC:10659]	0.044	-4.500629015
Ras association domain family member 2 [Source:HGNC Symbol;Acc:HGN	0.044	-4.500629015
long intergenic non-protein coding RNA 941 [Source:HGNC Symbol;Acc:H	0.024	-5.404002679
novel transcript	0.146	-2.772326656
novel transcript	0.475	-1.072664886
eukaryotic translation elongation factor 1 delta pseudogene 5 [Source:H	0.174	-2.521387575
KDM4A antisense RNA 1 [Source:HGNC Symbol;Acc:HGNC:40528]	2.346	1.229977398
C-type lectin domain containing 11A [Source:HGNC Symbol;Acc:HGNC:10	0.223	-2.164694062
CMT1A duplicated region transcript 4 [Source:HGNC Symbol;Acc:HGNC:1	21.367	4.417309886
neutrophil cytosolic factor 4 [Source:HGNC Symbol;Acc:HGNC:7662]	0.025	-5.298995154
NKD2, WNT signaling pathway inhibitor [Source:HGNC Symbol;Acc:HGNC	0.438	-1.189370663
SPECC1L-ADORA2A readthrough (NMD candidate) [Source:HGNC Symbo	0.136	-2.879965414
dedicator of cytokinesis 2 [Source:HGNC Symbol;Acc:HGNC:2988]	0.048	-4.369384482
erythrocyte membrane protein band 4.1 like 3 [Source:HGNC Symbol;Acc	0.048	-4.369384482
enolase 1 pseudogene 1 [Source:HGNC Symbol;Acc:HGNC:3352]	0.167	-2.578656349
Wnt family member 4 [Source:HGNC Symbol;Acc:HGNC:12783]	2.05	1.035532978
SFT2 domain containing 3 [Source:HGNC Symbol;Acc:HGNC:28767]	0.026	-5.264687103

novel transcript	0.254	-1.977067059
SATB homeobox 1 [Source:HGNC Symbol;Acc:HGNC:10541]	0.254	-1.977067059
zinc finger protein 554 [Source:HGNC Symbol;Acc:HGNC:26629]	0.428	-1.22509704
apolipoprotein C2 [Source:HGNC Symbol;Acc:HGNC:609]	0.027	-5.224994573
ribosomal protein S4 Y-linked 1 [Source:HGNC Symbol;Acc:HGNC:10425]	0.027	-5.224994573
leupaxin [Source:HGNC Symbol;Acc:HGNC:14061]	0.414	-1.272522943
keratin 23 [Source:HGNC Symbol;Acc:HGNC:6438]	0.414	-1.272522943
intercellular adhesion molecule 3 [Source:HGNC Symbol;Acc:HGNC:5346	0.319	-1.64924525
NCK associated protein 1 like [Source:HGNC Symbol;Acc:HGNC:4862]	0.088	-3.500629015
POU class 5 homeobox 1B [Source:HGNC Symbol;Acc:HGNC:9223]	0.051	-4.298995154
elastin microfibril interfacer 2 [Source:HGNC Symbol;Acc:HGNC:19881]	0.051	-4.298995154
NPHP3-ACAD11 readthrough (NMD candidate) [Source:HGNC Symbol;Ac	0.481	-1.054408756
RAB11B antisense RNA 1 [Source:HGNC Symbol;Acc:HGNC:44178]	0.499	-1.00215804
leukotriene B4 receptor 2 [Source:HGNC Symbol;Acc:HGNC:19260]	4.881	2.287319451
novel protein	0.281	-1.830347187
F-box protein 16 [Source:HGNC Symbol;Acc:HGNC:13618]	0.464	-1.108049916
spondin 2 [Source:HGNC Symbol;Acc:HGNC:11253]	2.171	1.118090174
ectonucleotide pyrophosphatase/phosphodiesterase 3 [Source:HGNC Sy	0.028	-5.161347354
C-type lectin domain family 18 member A [Source:HGNC Symbol;Acc:HG	0.283	-1.818657839
novel transcript	4.28	2.097750219
ATPase phospholipid transporting 10A (putative) [Source:HGNC Symbol;/	0.028	-5.146992061
interleukin 24 [Source:HGNC Symbol;Acc:HGNC:11346]	0.491	-1.026068449
protocadherin gamma subfamily B, 1 [Source:HGNC Symbol;Acc:HGNC:8	2.13	1.090582448
IKAROS family zinc finger 1 [Source:HGNC Symbol;Acc:HGNC:13176]	0.404	-1.308910623
glutamate metabotropic receptor 5 [Source:HGNC Symbol;Acc:HGNC:45	0.029	-5.13168267
novel transcript	10.515	3.394360297
adhesion molecule with Ig like domain 3 [Source:HGNC Symbol;Acc:HGN	2.702	1.434024526
IQ motif containing with AAA domain 1 [Source:HGNC Symbol;Acc:HGNC	2.322	1.215578019
thymic stromal lymphopoietin [Source:HGNC Symbol;Acc:HGNC:30743]	0.254	-1.977067059
novel transcript, antisense to SYNM	0.029	-5.107997929
kallikrein related peptidase 11 [Source:HGNC Symbol;Acc:HGNC:6359]	0.441	-1.182181489
eukaryotic translation elongation factor 1 gamma (	0.074	-3.763663421
ELOA antisense RNA 1 [Source:HGNC Symbol;Acc:HGNC:50582]	0.345	-1.535208538
olfactory receptor family 2 subfamily A member 9 pseudogene [Source:H	0.354	-1.497978012
RIMS binding protein 3C [Source:HGNC Symbol;Acc:HGNC:33892]	13.147	3.716608252
novel transcript, antisense to RIPK1	0.056	-4.146992061
DNAAF4-CCPG1 readthrough (NMD candidate) [Source:HGNC Symbol;Ac	33.102	5.04884764
solute carrier family 22 member 1 [Source:HGNC Symbol;Acc:HGNC:109	0.368	-1.442730632
novel transcript	0.031	-5.019711397
novel transcript, overlapping TTLL11	32.675	5.030128442
novel transcript, RBM7-REXO2 readthrough	32.635	5.028332929
novel transcript	0.363	-1.462493886
transmembrane protein 217 [Source:HGNC Symbol;Acc:HGNC:21238]	0.132	-2.919620532
Serine/threonine-protein kinase ULK4 (ULK4), pseudogene	0.031	-4.992314833
neuregulin 2 [Source:HGNC Symbol;Acc:HGNC:7998]	3.759	1.910458212
diacylglycerol kinase beta [Source:HGNC Symbol;Acc:HGNC:2850]	0.032	-4.977067059
novel transcript	0.034	-4.893904197

novel transcript	0.032	-4.96892899
novel transcript, antisense RAB8A	0.238	-2.072420292
RASD family member 2 [Source:HGNC Symbol;Acc:HGNC:18229]	2.632	1.396391336
phosphatase domain containing paladin 1 [Source:HGNC Symbol;Acc:HG	0.102	-3.298995154
novel protein	3.937	1.977025134
UL16 binding protein 1 [Source:HGNC Symbol;Acc:HGNC:14893]	0.389	-1.360395699
FAT atypical cadherin 3 [Source:HGNC Symbol;Acc:HGNC:23112]	0.473	-1.081403719
novel transcript	0.032	-4.947000334
novel transcript, antisense to ANKRD40	0.085	-3.550497164
BCL2 related protein A1 [Source:HGNC Symbol;Acc:HGNC:991]	0.06	-4.0645299
cell adhesion molecule L1 like [Source:HGNC Symbol;Acc:HGNC:1939]	0.06	-4.0645299
Spi-1 proto-oncogene [Source:HGNC Symbol;Acc:HGNC:11241]	0.06	-4.0645299
novel readthrough SHPK-TRPV1 protein	31.07	4.957449442
novel transcript, antisense to OS9	16.856	4.075174922
RAB11 family interacting protein 1 pseudogene 1 [Source:HGNC Symbol;	2.219	1.150221033
RPA1 related single stranded DNA binding protein, X-linked [Source:HGN	0.456	-1.133186261
limbic system associated membrane protein [Source:HGNC Symbol;Acc:H	0.21	-2.250085554
novel transcript	0.233	-2.102639512
novel protein	30.521	4.931745849
novel transcript, antisense to MORC3	0.139	-2.851536177
novel transcript, antisense to L3MBTL2	0.472	-1.082892162
MED14 opposite strand [Source:HGNC Symbol;Acc:HGNC:40162]	5.283	2.401444564
cytochrome P450 family 2 subfamily D member 6 [Source:HGNC Symbol;	2.758	1.463505532
ribosomal protein S15 (RPS15) pseudogene	9.557	3.256505161
SAM and SH3 domain containing 3 [Source:HGNC Symbol;Acc:HGNC:159	0.034	-4.883957655
membrane spanning 4-domains A6A [Source:HGNC Symbol;Acc:HGNC:1	0.034	-4.883957655
BAI1 associated protein 3 [Source:HGNC Symbol;Acc:HGNC:948]	2.727	1.447430769
novel transcript	0.188	-2.413917382
novel transcript, antisense to GNA11	0.034	-4.873338908
fucosyltransferase 7 [Source:HGNC Symbol;Acc:HGNC:4018]	0.034	-4.872369681
solute carrier family 2 member 5 [Source:HGNC Symbol;Acc:HGNC:1101	0.169	-2.56202956
periaxin [Source:HGNC Symbol;Acc:HGNC:13797]	2.312	1.209346065
sperm associated antigen 6 [Source:HGNC Symbol;Acc:HGNC:11215]	0.484	-1.047456387
RNA component of mitochondrial RNA processing endoribonuclease [Sou	0.035	-4.844963523
RNA, 7SK small nuclear [Source:HGNC Symbol;Acc:HGNC:10037]	0.461	-1.118422908
mitochondrially encoded cytochrome c oxidase II (MT-CO2) pseudogene	2.652	1.407285443
novel protein	0.035	-4.837036607
TNF superfamily member 14 [Source:HGNC Symbol;Acc:HGNC:11930]	2.269	1.182131536
cytohesin 4 [Source:HGNC Symbol;Acc:HGNC:9505]	0.331	-1.595976892
solute carrier family 25 member 21 [Source:HGNC Symbol;Acc:HGNC:14	0.183	-2.449880465
tubulin beta 2B class IIb [Source:HGNC Symbol;Acc:HGNC:30829]	0.377	-1.409012626
novel protein	0.036	-4.810969136
homeobox C5 [Source:HGNC Symbol;Acc:HGNC:5127]	28.449	4.830287863
novel protein	8.702	3.121354994
chromosome X open reading frame 49 (CXorf49) pseudogene	2.229	1.156199472
adhesion G protein-coupled receptor A2 [Source:HGNC Symbol;Acc:HGN	0.358	-1.481561026
novel transcript, antisense to C7orf49	6.49	2.698158892

inactivation escape 2 [Source:HGNC Symbol;Acc:HGNC:6061]	0.036	-4.796735243
natriuretic peptide receptor 3 [Source:HGNC Symbol;Acc:HGNC:7945]	2.709	1.43797044
platelet and endothelial cell adhesion molecule 1 [Source:HGNC Symbol;	0.036	-4.784421981
docking protein 2 [Source:HGNC Symbol;Acc:HGNC:2991]	0.036	-4.784421981
protein tyrosine phosphatase, non-receptor type 7 [Source:HGNC Symbc	0.036	-4.784421981
novel transcript	2.057	1.040841228
novel transcript	27.819	4.797983482
killer cell lectin like receptor C2 [Source:HGNC Symbol;Acc:HGNC:6375]	0.112	-3.154187562
novel transcript	15.24	3.929823536
paraoxonase 3 [Source:HGNC Symbol;Acc:HGNC:9206]	0.29	-1.784421981
ST6 N-acetylgalactosaminide alpha-2,6-sialyltransferase 5 [Source:HGNC	0.435	-1.199459481
C1q and TNF related 12 [Source:HGNC Symbol;Acc:HGNC:32308]	0.277	-1.851536177
MIR200C and MIR141 host gene [Source:HGNC Symbol;Acc:HGNC:53161	6.773	2.759898535

Pvalue	Padjust	Significant	Regulate	Cal27_NC	Cal27_OE	Cal27_NC	Cal27_OE
0	0	yes	down	1439.52	711.79	1439.52	711.79
0	0	yes	up	0.11	222.34	0.11	222.34
2.9738E-124	1.4193E-121	yes	up	0.49	14.53	0.49	14.53
2.2329E-111	9.8373E-109	yes	down	15.01	0.57	15.01	0.57
4.13997E-77	1.12909E-74	yes	down	17.44	6.41	17.44	6.41
7.17057E-71	1.55955E-68	yes	down	10.1	4.85	10.1	4.85
1.23472E-60	2.25692E-58	yes	down	8.46	3.89	8.46	3.89
5.30429E-51	8.21066E-49	yes	up	8.23	38.34	8.23	38.34
7.73738E-51	1.187E-48	yes	up	0.37	4.69	0.37	4.69
3.50772E-48	4.98097E-46	yes	down	32.05	13.68	32.05	13.68
7.43424E-37	7.25768E-35	yes	down	6.73	2.95	6.73	2.95
1.43337E-35	1.3241E-33	yes	down	74.21	31.41	74.21	31.41
1.727E-34	1.53748E-32	yes	down	3.43	0.83	3.43	0.83
8.54347E-32	6.95707E-30	yes	up	4.55	7.37	4.55	7.37
3.47804E-31	2.76665E-29	yes	down	21.62	10.25	21.62	10.25
1.48448E-24	8.94959E-23	yes	down	23.27	10.23	23.27	10.23
6.33462E-24	3.70209E-22	yes	down	1.86	0.11	1.86	0.11
1.63476E-23	9.30079E-22	yes	up	0.21	2.55	0.21	2.55
1.74099E-22	9.46637E-21	yes	down	4.56	0	4.56	0
1.74099E-22	9.46637E-21	yes	down	1.81	0	1.81	0
8.16612E-20	3.93026E-18	yes	up	1.18	11.31	1.18	11.31
6.54272E-19	2.8899E-17	yes	down	18	14.07	18	14.07
2.31793E-17	8.8899E-16	yes	up	0.5	1.71	0.5	1.71
4.31316E-17	1.62519E-15	yes	down	1.55	0.57	1.55	0.57
2.50175E-16	8.89962E-15	yes	down	5.98	3.02	5.98	3.02
4.70473E-16	1.62977E-14	yes	up	5.03	9.28	5.03	9.28
5.15489E-16	1.77305E-14	yes	up	0	2.77	0	2.77
1.09696E-15	3.66692E-14	yes	down	2.98	1.04	2.98	1.04
6.28814E-15	1.97519E-13	yes	up	7.13	28.78	7.13	28.78
8.6762E-15	2.70063E-13	yes	down	16.83	5.5	16.83	5.5
2.62738E-14	7.83745E-13	yes	down	3.19	1.51	3.19	1.51
5.19599E-14	1.49294E-12	yes	down	4.75	1.69	4.75	1.69
5.45731E-14	1.56279E-12	yes	up	0	2.41	0	2.41
1.76227E-12	4.33802E-11	yes	down	3.09	0.71	3.09	0.71
1.82954E-12	4.48432E-11	yes	down	48.69	21.23	48.69	21.23
2.19927E-12	5.29984E-11	yes	down	6.15	2.44	6.15	2.44
3.63191E-12	8.44431E-11	yes	up	0	2.25	0	2.25
5.00288E-12	1.15073E-10	yes	down	0.68	0	0.68	0
7.63512E-12	1.71486E-10	yes	down	2.01	0.52	2.01	0.52
9.45478E-12	2.1043E-10	yes	up	4.76	7.76	4.76	7.76
1.13283E-11	2.47952E-10	yes	up	8.65	25.84	8.65	25.84
2.08873E-11	4.45821E-10	yes	down	1	0.04	1	0.04
2.08873E-11	4.45821E-10	yes	down	2.42	0.1	2.42	0.1
2.61516E-11	5.5337E-10	yes	up	0	3.22	0	3.22
3.68243E-11	7.60475E-10	yes	down	1.05	0.09	1.05	0.09
6.50295E-11	1.30074E-09	yes	down	0.49	0.01	0.49	0.01

8.6046E-11	1.69741E-09 yes	up	5.05	11.71	5.05	11.71
1.1172E-10	2.17146E-09 yes	down	1.37	0	1.37	0
1.54964E-10	2.95515E-09 yes	down	9.43	4.36	9.43	4.36
2.31218E-10	4.30889E-09 yes	down	6.08	2.73	6.08	2.73
3.42549E-10	6.20197E-09 yes	down	3.1	0.96	3.1	0.96
3.71134E-10	6.67032E-09 yes	down	2.15	1.21	2.15	1.21
5.44176E-10	9.57995E-09 yes	down	2.59	0	2.59	0
5.44176E-10	9.57995E-09 yes	down	1.11	0	1.11	0
6.52022E-10	1.13621E-08 yes	down	0.69	0	0.69	0
7.11682E-10	1.23516E-08 yes	down	2.02	0.96	2.02	0.96
7.31708E-10	1.26736E-08 yes	down	1.7	0.36	1.7	0.36
1.08787E-09	1.83612E-08 yes	up	0.55	1.99	0.55	1.99
3.16889E-09	5.06022E-08 yes	down	1.36	0	1.36	0
3.195E-09	5.09717E-08 yes	up	0	0.63	0	0.63
3.35634E-09	5.32983E-08 yes	up	0	0.32	0	0.32
4.0927E-09	6.42786E-08 yes	down	3.8	1.72	3.8	1.72
6.55252E-09	9.95451E-08 yes	down	1.24	0.02	1.24	0.02
6.57909E-09	9.98604E-08 yes	up	0.97	2.33	0.97	2.33
9.6378E-09	1.4251E-07 yes	down	0.9	0	0.9	0
1.06039E-08	1.55457E-07 yes	down	1	0.16	1	0.16
1.28612E-08	1.87273E-07 yes	down	2.34	0.66	2.34	0.66
1.47155E-08	2.12472E-07 yes	down	0.83	0.25	0.83	0.25
2.10744E-08	2.97535E-07 yes	down	1.45	0.59	1.45	0.59
2.24374E-08	3.1471E-07 yes	down	2.81	0.86	2.81	0.86
2.74873E-08	3.80877E-07 yes	up	3.98	4.73	3.98	4.73
2.90271E-08	4.00276E-07 yes	up	0.51	2.45	0.51	2.45
3.36066E-08	4.58277E-07 yes	up	1.29	3.1	1.29	3.1
3.40234E-08	4.62859E-07 yes	down	1.01	0.08	1.01	0.08
3.88906E-08	5.23683E-07 yes	down	0.53	0.19	0.53	0.19
4.35495E-08	5.79155E-07 yes	up	2.01	3.07	2.01	3.07
4.40862E-08	5.85288E-07 yes	down	2.42	1.43	2.42	1.43
4.54339E-08	6.00035E-07 yes	up	0	5.1	0	5.1
5.75648E-08	7.44789E-07 yes	up	1.19	2.61	1.19	2.61
6.20355E-08	8.00823E-07 yes	up	0.03	0.34	0.03	0.34
9.71412E-08	1.22636E-06 yes	down	3.13	1.27	3.13	1.27
1.04648E-07	1.31342E-06 yes	up	0.99	11.68	0.99	11.68
1.24505E-07	1.54569E-06 yes	up	0.19	0.93	0.19	0.93
1.47111E-07	1.81455E-06 yes	up	0	1	0	1
1.82838E-07	2.21391E-06 yes	down	6.79	1.59	6.79	1.59
1.94903E-07	2.34676E-06 yes	up	0.5	1.46	0.5	1.46
1.97988E-07	2.37558E-06 yes	down	1.1	0	1.1	0
2.0249E-07	2.4279E-06 yes	up	0.9	2.38	0.9	2.38
2.17663E-07	2.59714E-06 yes	down	2.8	0.23	2.8	0.23
2.84918E-07	3.31223E-06 yes	down	4.23	1.97	4.23	1.97
3.2314E-07	3.71384E-06 yes	up	0.91	2.25	0.91	2.25
3.55913E-07	4.0365E-06 yes	down	0.62	0	0.62	0
3.89216E-07	4.38149E-06 yes	down	0.58	0.03	0.58	0.03

3.89647E-07	4.38149E-06 yes	down	0.71	0	0.71	0
3.89647E-07	4.38149E-06 yes	down	0.27	0	0.27	0
4.0961E-07	4.59095E-06 yes	down	0.44	0.02	0.44	0.02
4.0961E-07	4.59095E-06 yes	down	0.54	0.02	0.54	0.02
4.17109E-07	4.66891E-06 yes	up	1.8	6.37	1.8	6.37
4.33529E-07	4.83695E-06 yes	down	3.66	1.36	3.66	1.36
4.94453E-07	5.45997E-06 yes	down	1.4	0.16	1.4	0.16
5.05147E-07	5.55662E-06 yes	down	1.26	0.06	1.26	0.06
5.61514E-07	6.12179E-06 yes	up	0	1.76	0	1.76
5.79675E-07	6.30379E-06 yes	down	9.83	0	9.83	0
6.61081E-07	7.12145E-06 yes	down	5.54	2.97	5.54	2.97
6.79799E-07	7.29109E-06 yes	down	1.09	0.01	1.09	0.01
7.07496E-07	7.54575E-06 yes	down	0.97	0	0.97	0
7.10318E-07	7.56645E-06 yes	down	0.37	0.03	0.37	0.03
7.32925E-07	7.79278E-06 yes	up	2.76	3.68	2.76	3.68
7.45485E-07	7.91163E-06 yes	down	0.22	0.01	0.22	0.01
7.49498E-07	7.94441E-06 yes	up	0	2.07	0	2.07
7.74209E-07	8.16603E-06 yes	up	19.34	129.09	19.34	129.09
8.40178E-07	8.75968E-06 yes	up	0.36	1.2	0.36	1.2
8.69581E-07	9.01699E-06 yes	down	1.32	0.56	1.32	0.56
8.99131E-07	9.29535E-06 yes	down	0.62	0	0.62	0
9.35698E-07	9.64437E-06 yes	up	2.34	4.6	2.34	4.6
9.44684E-07	9.71369E-06 yes	down	4.07	1.56	4.07	1.56
1.0248E-06	1.04873E-05 yes	down	0.23	0.01	0.23	0.01
1.21782E-06	1.2251E-05 yes	down	1.68	0	1.68	0
1.21782E-06	1.2251E-05 yes	down	1.54	0	1.54	0
1.27502E-06	1.27517E-05 yes	down	0.45	0.11	0.45	0.11
1.28066E-06	1.27858E-05 yes	up	0.68	3.03	0.68	3.03
1.32142E-06	1.31698E-05 yes	down	9.25	4.14	9.25	4.14
1.32876E-06	1.32353E-05 yes	down	1.16	0.02	1.16	0.02
1.35959E-06	1.35266E-05 yes	down	0.44	0.02	0.44	0.02
1.35959E-06	1.35266E-05 yes	down	0.24	0.01	0.24	0.01
1.61364E-06	1.57985E-05 yes	down	1.08	0	1.08	0
2.12928E-06	2.0496E-05 yes	down	0.31	0.04	0.31	0.04
2.4104E-06	2.29449E-05 yes	down	6.07	4.45	6.07	4.45
2.5451E-06	2.41469E-05 yes	down	3.85	0.65	3.85	0.65
2.84639E-06	2.67542E-05 yes	up	5.02	13.04	5.02	13.04
3.31631E-06	3.0734E-05 yes	down	2.05	0.44	2.05	0.44
3.54454E-06	3.24985E-05 yes	up	0.15	1.48	0.15	1.48
3.86327E-06	3.51211E-05 yes	down	0.89	0	0.89	0
4.11787E-06	3.71018E-05 yes	down	3.84	1.62	3.84	1.62
4.51153E-06	4.02687E-05 yes	down	0.37	0.03	0.37	0.03
4.55063E-06	4.05545E-05 yes	down	0.23	0.18	0.23	0.18
4.55063E-06	4.05545E-05 yes	down	0.5	0.08	0.5	0.08
4.74671E-06	4.20836E-05 yes	down	1.7	0.28	1.7	0.28
4.94899E-06	4.37191E-05 yes	up	1.44	3.75	1.44	3.75
5.07887E-06	4.47056E-05 yes	down	0.3	0	0.3	0

5.48906E-06	4.78746E-05	yes	down	1.8	0.45	1.8	0.45
5.48906E-06	4.78746E-05	yes	down	0.62	0.34	0.62	0.34
5.69549E-06	4.94742E-05	yes	down	1.92	0.79	1.92	0.79
6.92175E-06	5.89344E-05	yes	down	2.49	0	2.49	0
6.92175E-06	5.89344E-05	yes	down	1.34	0	1.34	0
7.30174E-06	6.19549E-05	yes	down	2.81	1.14	2.81	1.14
7.30174E-06	6.19549E-05	yes	down	3	1.27	3	1.27
7.62681E-06	6.44555E-05	yes	down	1.89	1.28	1.89	1.28
7.88035E-06	6.63727E-05	yes	down	0.71	0.15	0.71	0.15
8.35165E-06	6.96931E-05	yes	down	0.48	0.03	0.48	0.03
8.35165E-06	6.96931E-05	yes	down	0.32	0.01	0.32	0.01
9.44661E-06	7.80345E-05	yes	down	7.25	3.1	7.25	3.1
9.45287E-06	7.80487E-05	yes	down	5.59	3.6	5.59	3.6
9.60884E-06	7.91843E-05	yes	up	0.36	0.65	0.36	0.65
1.02746E-05	8.39459E-05	yes	down	1.88	0.51	1.88	0.51
1.0832E-05	8.79563E-05	yes	down	5.27	3.8	5.27	3.8
1.10871E-05	8.96885E-05	yes	up	2.54	5.01	2.54	5.01
1.12004E-05	9.05203E-05	yes	down	4.15	0	4.15	0
1.153E-05	9.29218E-05	yes	down	0.92	0.28	0.92	0.28
1.20209E-05	9.65501E-05	yes	up	0.43	1.79	0.43	1.79
1.24533E-05	9.97076E-05	yes	down	0.15	0	0.15	0
1.26659E-05	0.000101221	yes	down	4.79	2.62	4.79	2.62
1.30739E-05	0.000104095	yes	up	0.68	1.23	0.68	1.23
1.35092E-05	0.000107262	yes	down	2.44	0.32	2.44	0.32
1.39302E-05	0.000110248	yes	down	0.23	0	0.23	0
1.39411E-05	0.000110284	yes	up	0.06	0.65	0.06	0.65
1.44897E-05	0.000113898	yes	up	0.49	1.28	0.49	1.28
1.46416E-05	0.000114926	yes	up	0.64	1.71	0.64	1.71
1.46657E-05	0.00011501	yes	down	1.01	0.25	1.01	0.25
1.65341E-05	0.000128315	yes	down	0.41	0	0.41	0
1.83582E-05	0.000141069	yes	down	4.93	2.67	4.93	2.67
1.85753E-05	0.000142674	yes	down	1.04	0.07	1.04	0.07
2.08407E-05	0.000158375	yes	down	3.73	1.25	3.73	1.25
2.53999E-05	0.000189419	yes	down	1.61	0.71	1.61	0.71
2.63261E-05	0.000195731	yes	up	0.02	0.2	0.02	0.2
2.83077E-05	0.000208838	yes	down	1.71	0.09	1.71	0.09
2.90818E-05	0.000214181	yes	up	0	0.2	0	0.2
2.94752E-05	0.000216892	yes	down	2.08	1.39	2.08	1.39
3.06635E-05	0.000223952	yes	down	0.28	0	0.28	0
3.30229E-05	0.000239409	yes	up	0	0.24	0	0.24
3.34254E-05	0.000242021	yes	up	0	1.92	0	1.92
3.37111E-05	0.000243576	yes	down	3.13	1.1	3.13	1.1
3.56418E-05	0.000255805	yes	down	0.71	0.1	0.71	0.1
3.68973E-05	0.000264044	yes	down	6.97	0	6.97	0
4.01147E-05	0.000284579	yes	up	0.57	0.82	0.57	0.82
4.08464E-05	0.000288697	yes	down	0.13	0	0.13	0
4.24136E-05	0.00029818	yes	down	1.96	0.06	1.96	0.06

4.31078E-05	0.000302728	yes	down	0.23	0	0.23	0
4.3765E-05	0.000306865	yes	down	1.74	0.15	1.74	0.15
4.64399E-05	0.000323573	yes	up	0.37	0.96	0.37	0.96
4.77374E-05	0.000330602	yes	down	0.25	0.02	0.25	0.02
4.80364E-05	0.000332004	yes	up	0.2	0.78	0.2	0.78
4.82408E-05	0.000333015	yes	down	1.1	0.42	1.1	0.42
4.92315E-05	0.000338765	yes	down	2.29	0.4	2.29	0.4
4.97786E-05	0.000342119	yes	down	3.86	0	3.86	0
5.1159E-05	0.000350344	yes	down	2.59	0.22	2.59	0.22
5.22787E-05	0.000356874	yes	down	1.84	0.11	1.84	0.11
5.22787E-05	0.000356874	yes	down	0.27	0.01	0.27	0.01
5.22787E-05	0.000356874	yes	down	1.35	0.05	1.35	0.05
5.33693E-05	0.000363384	yes	up	0	0.18	0	0.18
5.55128E-05	0.000376261	yes	up	0.13	2.12	0.13	2.12
5.63759E-05	0.00038196	yes	up	1.26	2.71	1.26	2.71
5.95059E-05	0.000402057	yes	down	1.69	0.77	1.69	0.77
6.18936E-05	0.0004159	yes	down	0.26	0.04	0.26	0.04
6.19832E-05	0.000416339	yes	down	0.31	0.07	0.31	0.07
6.29295E-05	0.000422365	yes	up	0	0.43	0	0.43
6.78619E-05	0.000452305	yes	down	1.98	0.27	1.98	0.27
6.93388E-05	0.000461239	yes	down	2.25	0.92	2.25	0.92
7.05454E-05	0.000468177	yes	up	0.44	2.24	0.44	2.24
7.09809E-05	0.000470341	yes	up	0.66	1.52	0.66	1.52
7.10363E-05	0.000470527	yes	up	0.22	2.07	0.22	2.07
7.44949E-05	0.000490035	yes	down	0.34	0	0.34	0
7.44949E-05	0.000490035	yes	down	2.36	0	2.36	0
7.55207E-05	0.000496024	yes	up	0.39	3.42	0.39	3.42
7.68114E-05	0.000502771	yes	down	3.98	0.51	3.98	0.51
7.96092E-05	0.000519107	yes	down	4.67	0	4.67	0
8.00914E-05	0.000521855	yes	down	0.35	0	0.35	0
8.47006E-05	0.000548352	yes	down	0.35	0.18	0.35	0.18
8.83246E-05	0.000568813	yes	up	0.33	0.75	0.33	0.75
9.27724E-05	0.000595004	yes	down	2.13	1	2.13	1
9.48666E-05	0.00060685	yes	down	28.96	0	28.96	0
9.8012E-05	0.000624182	yes	down	63.46	29.03	63.46	29.03
9.88228E-05	0.00062888	yes	up	1.76	4.56	1.76	4.56
9.95755E-05	0.000632264	yes	down	0.32	0	0.32	0
0.000104645	0.000658614	yes	up	0.44	1.16	0.44	1.16
0.000106736	0.000670299	yes	down	2.08	0.9	2.08	0.9
0.000112544	0.000703689	yes	down	2.75	0.11	2.75	0.11
0.000115223	0.000717356	yes	down	29.28	8.09	29.28	8.09
0.000116581	0.000724709	yes	down	0.27	0	0.27	0
0.000117673	0.000729915	yes	up	0	0.49	0	0.49
0.000119812	0.000740774	yes	up	0.24	2.05	0.24	2.05
0.000123092	0.000759415	yes	up	7	15.26	7	15.26
0.000123685	0.000762524	yes	down	0.47	0.15	0.47	0.15
0.000124788	0.0007685	yes	up	0.21	1.31	0.21	1.31

0.000126929	0.000780285	yes	down	0.48	0	0.48	0
0.000132822	0.000813309	yes	up	0.44	0.53	0.44	0.53
0.000136538	0.000833096	yes	down	1.04	0	1.04	0
0.000136538	0.000833096	yes	down	0.68	0	0.68	0
0.000136538	0.000833096	yes	down	0.27	0	0.27	0
0.000138024	0.000841267	yes	up	0.42	1.64	0.42	1.64
0.000142484	0.000864465	yes	up	0	0.3	0	0.3
0.000149682	0.000902718	yes	down	1.3	0.35	1.3	0.35
0.000150256	0.000905542	yes	up	0.01	0.2	0.01	0.2
0.000154973	0.000930053	yes	down	2.04	0.62	2.04	0.62
0.00015966	0.000955846	yes	down	1.92	0.9	1.92	0.9
0.000164973	0.000984569	yes	down	4.77	1.39	4.77	1.39
0.000166645	0.000993168	yes	up	2.01	13.46	2.01	13.46

Interferon-inducible protein, IFIX, has tumor-suppressive effects in oral squamous cell carcinoma

Shan Wang, PhD , Fang Li, M.M , Haixia Fan, PhD

Supplementary data 3 showed the set of 248 genes/RNAs for enrichment with potential CSC signals by KEGG analysis

<label class	Pathway id	Description	Database
7	map04514	Cell adhesion molecules (CAMs)	KEGG PATHWAY
5	map04725	Cholinergic synapse	KEGG PATHWAY
4	map05221	Acute myeloid leukemia	KEGG PATHWAY
4	map04911	Insulin secretion	KEGG PATHWAY
6	map04062	Chemokine signaling pathway	KEGG PATHWAY
3	map04927	Cortisol synthesis and secretion	KEGG PATHWAY
13	map05200	Pathways in cancer	KEGG PATHWAY
4	map04540	Gap junction	KEGG PATHWAY
8	map05202	Transcriptional misregulation in cancer	KEGG PATHWAY
6	map05163	Human cytomegalovirus infection	KEGG PATHWAY
4	map04660	T cell receptor signaling pathway	KEGG PATHWAY
3	map04970	Salivary secretion	KEGG PATHWAY
4	map04926	Relaxin signaling pathway	KEGG PATHWAY
3	map05144	Malaria	KEGG PATHWAY
3	map04750	Inflammatory mediator regulation of TRP channels	KEGG PATHWAY
5	map04650	Natural killer cell mediated cytotoxicity	KEGG PATHWAY
2	map04973	Carbohydrate digestion and absorption	KEGG PATHWAY
3	map04925	Aldosterone synthesis and secretion	KEGG PATHWAY
3	map04668	TNF signaling pathway	KEGG PATHWAY
3	map04350	TGF-beta signaling pathway	KEGG PATHWAY
3	map04724	Glutamatergic synapse	KEGG PATHWAY
3	map04070	Phosphatidylinositol signaling system	KEGG PATHWAY
5	map04060	Cytokine-cytokine receptor interaction	KEGG PATHWAY
6	map04670	Leukocyte transendothelial migration	KEGG PATHWAY
3	map05231	Choline metabolism in cancer	KEGG PATHWAY
3	map04726	Serotonergic synapse	KEGG PATHWAY
3	map05211	Renal cell carcinoma	KEGG PATHWAY
5	map04934	Cushing syndrome	KEGG PATHWAY
3	map04928	Parathyroid hormone synthesis, secretion and actio	KEGG PATHWAY
5	map04072	Phospholipase D signaling pathway	KEGG PATHWAY
4	map04024	cAMP signaling pathway	KEGG PATHWAY
2	map04730	Long-term depression	KEGG PATHWAY
3	map04914	Progesterone-mediated oocyte maturation	KEGG PATHWAY
3	map04152	AMPK signaling pathway	KEGG PATHWAY
3	map04728	Dopaminergic synapse	KEGG PATHWAY
3	map04071	Sphingolipid signaling pathway	KEGG PATHWAY
3	map04380	Osteoclast differentiation	KEGG PATHWAY
4	map04022	cGMP-PKG signaling pathway	KEGG PATHWAY
2	map04918	Thyroid hormone synthesis	KEGG PATHWAY
3	map04270	Vascular smooth muscle contraction	KEGG PATHWAY
4	map04915	Estrogen signaling pathway	KEGG PATHWAY
1	map00604	Glycosphingolipid biosynthesis - ganglio series	KEGG PATHWAY
4	map04666	Fc gamma R-mediated phagocytosis	KEGG PATHWAY
2	map04720	Long-term potentiation	KEGG PATHWAY
1	map00770	Pantothenate and CoA biosynthesis	KEGG PATHWAY
3	map05142	Chagas disease (American trypanosomiasis)	KEGG PATHWAY

4	map04080	Neuroactive ligand-receptor interaction	KEGG PATHWAY
1	map00740	Riboflavin metabolism	KEGG PATHWAY
4	map04064	NF-kappa B signaling pathway	KEGG PATHWAY
4	map04550	Signaling pathways regulating pluripotency of stem	KEGG PATHWAY
3	map04630	Jak-STAT signaling pathway	KEGG PATHWAY
5	map05166	Human T-cell leukemia virus 1 infection	KEGG PATHWAY
2	map05218	Melanoma	KEGG PATHWAY
2	map05230	Central carbon metabolism in cancer	KEGG PATHWAY
2	map05133	Pertussis	KEGG PATHWAY
4	map05226	Gastric cancer	KEGG PATHWAY
1	map00531	Glycosaminoglycan degradation	KEGG PATHWAY
3	map05222	Small cell lung cancer	KEGG PATHWAY
4	map04310	Wnt signaling pathway	KEGG PATHWAY
7	map05016	Huntington disease	KEGG PATHWAY
4	map05146	Amoebiasis	KEGG PATHWAY
6	map04810	Regulation of actin cytoskeleton	KEGG PATHWAY
4	map05224	Breast cancer	KEGG PATHWAY
6	map04510	Focal adhesion	KEGG PATHWAY
2	map04713	Circadian entrainment	KEGG PATHWAY
5	map04020	Calcium signaling pathway	KEGG PATHWAY
2	map04620	Toll-like receptor signaling pathway	KEGG PATHWAY
2	map04912	GnRH signaling pathway	KEGG PATHWAY
2	map04211	Longevity regulating pathway	KEGG PATHWAY
3	map04360	Axon guidance	KEGG PATHWAY
2	map04931	Insulin resistance	KEGG PATHWAY
7	map04151	PI3K-Akt signaling pathway	KEGG PATHWAY
1	map00601	Glycosphingolipid biosynthesis - lacto and neolacto	KEGG PATHWAY
3	map05161	Hepatitis B	KEGG PATHWAY
2	map04012	ErbB signaling pathway	KEGG PATHWAY
2	map00564	Glycerophospholipid metabolism	KEGG PATHWAY
2	map04974	Protein digestion and absorption	KEGG PATHWAY
2	map01522	Endocrine resistance	KEGG PATHWAY
3	map04066	HIF-1 signaling pathway	KEGG PATHWAY
4	map04150	mTOR signaling pathway	KEGG PATHWAY
2	map05215	Prostate cancer	KEGG PATHWAY
3	map04916	Melanogenesis	KEGG PATHWAY
2	map04972	Pancreatic secretion	KEGG PATHWAY
2	map00980	Metabolism of xenobiotics by cytochrome P450	KEGG PATHWAY
1	map00500	Starch and sucrose metabolism	KEGG PATHWAY
1	map04392	Hippo signaling pathway - multiple species	KEGG PATHWAY
2	map04512	ECM-receptor interaction	KEGG PATHWAY
4	map04919	Thyroid hormone signaling pathway	KEGG PATHWAY
1	map00760	Nicotinate and nicotinamide metabolism	KEGG PATHWAY
2	map04922	Glucagon signaling pathway	KEGG PATHWAY
2	map04933	AGE-RAGE signaling pathway in diabetic complicatic	KEGG PATHWAY
1	map04960	Aldosterone-regulated sodium reabsorption	KEGG PATHWAY
2	map01521	EGFR tyrosine kinase inhibitor resistance	KEGG PATHWAY

6	map05165	Human papillomavirus infection	KEGG PATHWAY
4	map04723	Retrograde endocannabinoid signaling	KEGG PATHWAY
1	map05030	Cocaine addiction	KEGG PATHWAY
4	map04014	Ras signaling pathway	KEGG PATHWAY
1	map04962	Vasopressin-regulated water reabsorption	KEGG PATHWAY
1	map04930	Type II diabetes mellitus	KEGG PATHWAY
2	map05206	MicroRNAs in cancer	KEGG PATHWAY
5	map04015	Rap1 signaling pathway	KEGG PATHWAY
1	map00020	Citrate cycle (TCA cycle)	KEGG PATHWAY
1	map04961	Endocrine and other factor-regulated calcium reabs	KEGG PATHWAY
1	map04923	Regulation of lipolysis in adipocytes	KEGG PATHWAY
2	map04261	Adrenergic signaling in cardiomyocytes	KEGG PATHWAY
1	map00565	Ether lipid metabolism	KEGG PATHWAY
3	map03018	RNA degradation	KEGG PATHWAY
1	map04330	Notch signaling pathway	KEGG PATHWAY
1	map05031	Amphetamine addiction	KEGG PATHWAY
2	map05217	Basal cell carcinoma	KEGG PATHWAY
1	map04370	VEGF signaling pathway	KEGG PATHWAY
4	map04530	Tight junction	KEGG PATHWAY
3	map05203	Viral carcinogenesis	KEGG PATHWAY
1	map04976	Bile secretion	KEGG PATHWAY
3	map05168	Herpes simplex infection	KEGG PATHWAY
1	map04924	Renin secretion	KEGG PATHWAY
2	map04114	Oocyte meiosis	KEGG PATHWAY
1	map04917	Prolactin signaling pathway	KEGG PATHWAY
2	map04662	B cell receptor signaling pathway	KEGG PATHWAY
2	map04664	Fc epsilon RI signaling pathway	KEGG PATHWAY
1	map00561	Glycerolipid metabolism	KEGG PATHWAY
1	map04971	Gastric acid secretion	KEGG PATHWAY
1	map05213	Endometrial cancer	KEGG PATHWAY
1	map05223	Non-small cell lung cancer	KEGG PATHWAY
1	map00562	Inositol phosphate metabolism	KEGG PATHWAY
1	map04978	Mineral absorption	KEGG PATHWAY
2	map04612	Antigen processing and presentation	KEGG PATHWAY
1	map04213	Longevity regulating pathway - multiple species	KEGG PATHWAY
4	map04932	Non-alcoholic fatty liver disease (NAFLD)	KEGG PATHWAY
2	map04621	NOD-like receptor signaling pathway	KEGG PATHWAY
1	map00330	Arginine and proline metabolism	KEGG PATHWAY
5	map05205	Proteoglycans in cancer	KEGG PATHWAY
1	map05032	Morphine addiction	KEGG PATHWAY
2	map05340	Primary immunodeficiency	KEGG PATHWAY
1	map05214	Glioma	KEGG PATHWAY
1	map04727	GABAergic synapse	KEGG PATHWAY
3	map04611	Platelet activation	KEGG PATHWAY
1	map00140	Steroid hormone biosynthesis	KEGG PATHWAY
2	map04640	Hematopoietic cell lineage	KEGG PATHWAY
2	map05034	Alcoholism	KEGG PATHWAY

1	map00830	Retinol metabolism	KEGG PATHWAY
1	map05212	Pancreatic cancer	KEGG PATHWAY
1	map03320	PPAR signaling pathway	KEGG PATHWAY
3	map04144	Endocytosis	KEGG PATHWAY
2	map04141	Protein processing in endoplasmic reticulum	KEGG PATHWAY
3	map04010	MAPK signaling pathway	KEGG PATHWAY
2	map05167	Kaposi sarcoma-associated herpesvirus infection	KEGG PATHWAY
1	map04625	C-type lectin receptor signaling pathway	KEGG PATHWAY
1	map00982	Drug metabolism - cytochrome P450	KEGG PATHWAY
1	map01524	Platinum drug resistance	KEGG PATHWAY
3	map05418	Fluid shear stress and atherosclerosis	KEGG PATHWAY
1	map05220	Chronic myeloid leukemia	KEGG PATHWAY
1	map00240	Pyrimidine metabolism	KEGG PATHWAY
1	map05160	Hepatitis C	KEGG PATHWAY
4	map05010	Alzheimer disease	KEGG PATHWAY
1	map04658	Th1 and Th2 cell differentiation	KEGG PATHWAY
3	map04210	Apoptosis	KEGG PATHWAY
1	map05210	Colorectal cancer	KEGG PATHWAY
1	map05143	African trypanosomiasis	KEGG PATHWAY
1	map03008	Ribosome biogenesis in eukaryotes	KEGG PATHWAY
1	map04212	Longevity regulating pathway - worm	KEGG PATHWAY
1	map04013	MAPK signaling pathway - fly	KEGG PATHWAY
1	map00010	Glycolysis / Gluconeogenesis	KEGG PATHWAY
2	map05130	Pathogenic Escherichia coli infection	KEGG PATHWAY
1	map04659	Th17 cell differentiation	KEGG PATHWAY
1	map04722	Neurotrophin signaling pathway	KEGG PATHWAY
1	map04142	Lysosome	KEGG PATHWAY
2	map03013	RNA transport	KEGG PATHWAY
3	map04390	Hippo signaling pathway	KEGG PATHWAY
1	map04068	FoxO signaling pathway	KEGG PATHWAY
2	map04921	Oxytocin signaling pathway	KEGG PATHWAY
3	map05225	Hepatocellular carcinoma	KEGG PATHWAY
4	map05012	Parkinson disease	KEGG PATHWAY
1	map05145	Toxoplasmosis	KEGG PATHWAY
1	map05140	Leishmaniasis	KEGG PATHWAY
1	map05134	Legionellosis	KEGG PATHWAY
1	map04910	Insulin signaling pathway	KEGG PATHWAY
1	map04218	Cellular senescence	KEGG PATHWAY
1	map05150	Staphylococcus aureus infection	KEGG PATHWAY
1	map04745	Phototransduction - fly	KEGG PATHWAY
1	map00230	Purine metabolism	KEGG PATHWAY
2	map04140	Autophagy - animal	KEGG PATHWAY
2	map05169	Epstein-Barr virus infection	KEGG PATHWAY
3	map00190	Oxidative phosphorylation	KEGG PATHWAY
1	map05323	Rheumatoid arthritis	KEGG PATHWAY
1	map04260	Cardiac muscle contraction	KEGG PATHWAY
1	map05131	Shigellosis	KEGG PATHWAY

1	map04391	Hippo signaling pathway - fly	KEGG PATHWAY
1	map05132	Salmonella infection	KEGG PATHWAY
1	map05162	Measles	KEGG PATHWAY
1	map05100	Bacterial invasion of epithelial cells	KEGG PATHWAY
4	map04714	Thermogenesis	KEGG PATHWAY
1	map04217	Necroptosis	KEGG PATHWAY
2	map04145	Phagosome	KEGG PATHWAY
1	map05164	Influenza A	KEGG PATHWAY
2	map04740	Olfactory transduction	KEGG PATHWAY
1	map05416	Viral myocarditis	KEGG PATHWAY
2	map03010	Ribosome	KEGG PATHWAY

Ratio_in_st	Ratio_in_p	Pvalue_uncorrected	Pvalue_corrected	First Category	Second Cat
7/159	271/28425	0.000863291	0.170931698	Environmental Information	Signaling m
5/159	153/28425	0.001730004	0.171270369	Organismal Systems	Nervous sy
4/159	99/28425	0.002349716	0.155081274	Human Diseases	Cancers: Sp
4/159	110/28425	0.003437092	0.170136058	Organismal Systems	Endocrine s
6/159	274/28425	0.004542033	0.179864526	Organismal Systems	Immune sy
3/159	91/28425	0.014572327	0.480886799	Organismal Systems	Endocrine s
13/159	1177/2842	0.015186737	0.429567708	Human Diseases	Cancers: Ov
4/159	183/28425	0.019711861	0.487868559	Cellular Processes	Cellular cor
8/159	615/28425	0.022753373	0.500574199	Human Diseases	Cancers: Ov
6/159	417/28425	0.030254788	0.599044795	Human Diseases	Infectious d
4/159	211/28425	0.031086316	0.559553695	Organismal Systems	Immune sy
3/159	124/28425	0.032586736	0.53768114	Organismal Systems	Digestive sy
4/159	228/28425	0.039540963	0.602239286	Organismal Systems	Endocrine s
3/159	134/28425	0.039588433	0.559893551	Human Diseases	Infectious d
3/159	138/28425	0.042585751	0.562131908	Organismal Systems	Sensory sys
5/159	343/28425	0.044083928	0.545538609	Organismal Systems	Immune sy
2/159	60/28425	0.044554138	0.518924667	Organismal Systems	Digestive sy
3/159	141/28425	0.044906473	0.493971203	Organismal Systems	Endocrine s
3/159	145/28425	0.048096754	0.501218801	Environmental Information	Signal trans
3/159	146/28425	0.048911326	0.484222126	Environmental Information	Signal trans
3/159	152/28425	0.053939872	0.508575934	Organismal Systems	Nervous sy
3/159	152/28425	0.053939872	0.508575934	Environmental Information	Signal trans
5/159	365/28425	0.054789659	0.471667499	Environmental Information	Signaling m
6/159	485/28425	0.05566155	0.45920779	Organismal Systems	Immune sy
3/159	165/28425	0.065644879	0.519907439	Human Diseases	Cancers: Ov
3/159	167/28425	0.067541257	0.514352653	Organismal Systems	Nervous sy
3/159	167/28425	0.067541257	0.514352653	Human Diseases	Cancers: Sp
5/159	390/28425	0.068631554	0.485323135	Human Diseases	Endocrine a
3/159	173/28425	0.073378627	0.500998904	Organismal Systems	Endocrine s
5/159	403/28425	0.076529435	0.505094271	Environmental Information	Signal trans
4/159	289/28425	0.079578706	0.508276896	Environmental Information	Signal trans
2/159	85/28425	0.082184317	0.508515461	Organismal Systems	Nervous sy
3/159	185/28425	0.085698614	0.514191682	Organismal Systems	Endocrine s
3/159	187/28425	0.087832652	0.511496032	Environmental Information	Signal trans
3/159	188/28425	0.088908075	0.502965682	Organismal Systems	Nervous sy
3/159	188/28425	0.088908075	0.502965682	Environmental Information	Signal trans
3/159	190/28425	0.091075563	0.487377339	Organismal Systems	Developme
4/159	308/28425	0.095026429	0.495137709	Environmental Information	Signal trans
2/159	97/28425	0.102725499	0.521529455	Organismal Systems	Endocrine s
3/159	204/28425	0.106847833	0.528896771	Organismal Systems	Circulatory
4/159	325/28425	0.109955093	0.531002646	Organismal Systems	Endocrine s
1/159	22/28425	0.116135839	0.547497525	Metabolism	Glycan bios
4/159	332/28425	0.11639116	0.535940688	Organismal Systems	Immune sy
2/159	106/28425	0.118948842	0.535269791	Organismal Systems	Nervous sy
1/159	23/28425	0.12108371	0.532768326	Metabolism	Metabolism
3/159	216/28425	0.121152039	0.521480515	Human Diseases	Infectious d

4/159	342/28425	0.125864831	0.530239074	Environmental Information	Signaling m
1/159	24/28425	0.126004057	0.519766736	Metabolism	Metabolism
4/159	356/28425	0.139654735	0.564319131	Environmental Information	Signal trans
4/159	363/28425	0.146768632	0.581203783	Cellular Processes	Cellular cor
3/159	237/28425	0.147735496	0.573561336	Environmental Information	Signal trans
5/159	502/28425	0.151373708	0.576384502	Human Diseases	Infectious d
2/159	124/28425	0.153051145	0.571775976	Human Diseases	Cancers: Sp
2/159	126/28425	0.15695177	0.575489822	Human Diseases	Cancers: Ov
2/159	128/28425	0.160871202	0.579136326	Human Diseases	Infectious d
4/159	384/28425	0.168920896	0.597256026	Human Diseases	Cancers: Sp
1/159	33/28425	0.169073388	0.587307558	Metabolism	Glycan bios
3/159	253/28425	0.16914053	0.577410774	Human Diseases	Cancers: Sp
4/159	386/28425	0.171090298	0.57416744	Environmental Information	Signal trans
7/159	815/28425	0.173257571	0.571749984	Human Diseases	Neurodege
4/159	389/28425	0.174362778	0.565964426	Human Diseases	Infectious d
6/159	671/28425	0.174898862	0.558547979	Cellular Processes	Cell motilit
4/159	393/28425	0.178759685	0.561816152	Human Diseases	Cancers: Sp
6/159	676/28425	0.179006043	0.553799947	Cellular Processes	Cellular cor
2/159	140/28425	0.184733731	0.562727365	Organismal Systems	Environme
5/159	542/28425	0.188034455	0.564103366	Environmental Information	Signal trans
2/159	142/28425	0.18876066	0.557830011	Organismal Systems	Immune sy
2/159	145/28425	0.194823512	0.567280227	Organismal Systems	Endocrine s
2/159	146/28425	0.19685007	0.564874115	Organismal Systems	Aging
3/159	275/28425	0.199903956	0.565442617	Organismal Systems	Developme
2/159	148/28425	0.200911037	0.560287118	Human Diseases	Endocrine a
7/159	855/28425	0.203658762	0.560061596	Environmental Information	Signal trans
1/159	41/28425	0.205582888	0.557608382	Metabolism	Glycan bios
3/159	280/28425	0.207076808	0.554070379	Human Diseases	Infectious d
2/159	152/28425	0.209061822	0.55192321	Environmental Information	Signal trans
2/159	154/28425	0.213150215	0.555312403	Metabolism	Lipid metal
2/159	155/28425	0.215197358	0.553364635	Organismal Systems	Digestive sy
2/159	156/28425	0.217246351	0.551471506	Human Diseases	Drug resist
3/159	290/28425	0.22159234	0.555383334	Environmental Information	Signal trans
4/159	433/28425	0.224607038	0.555902418	Environmental Information	Signal trans
2/159	161/28425	0.227516158	0.556150607	Human Diseases	Cancers: Sp
3/159	298/28425	0.233349577	0.563453857	Organismal Systems	Endocrine s
2/159	165/28425	0.23575696	0.562408171	Organismal Systems	Digestive sy
2/159	166/28425	0.237819973	0.56057565	Metabolism	Xenobiotics
1/159	49/28425	0.24049787	0.560218569	Metabolism	Carbohydra
1/159	50/28425	0.244753609	0.563502495	Environmental Information	Signal trans
2/159	175/28425	0.256422006	0.583581117	Environmental Information	Signaling m
4/159	463/28425	0.260793013	0.58678428	Organismal Systems	Endocrine s
1/159	54/28425	0.261540899	0.581855035	Metabolism	Metabolism
2/159	178/28425	0.262631086	0.577788389	Organismal Systems	Endocrine s
2/159	180/28425	0.266771207	0.580447242	Human Diseases	Endocrine a
1/159	56/28425	0.269794964	0.580645684	Organismal Systems	Excretory s
2/159	182/28425	0.270911305	0.576778907	Human Diseases	Drug resist

6/159	793/28425	0.284478843	0.599221393	Human Diseases	Infectious (
4/159	493/28425	0.298020063	0.621136553	Organismal Systems	Nervous sy
1/159	64/28425	0.301904436	0.6226779	Human Diseases	Substance (
4/159	502/28425	0.309326129	0.631407974	Environmental Information	Signal trans
1/159	71/28425	0.328846834	0.664404827	Organismal Systems	Excretory s
1/159	71/28425	0.328846834	0.664404827	Human Diseases	Endocrine a
2/159	213/28425	0.33472734	0.662760133	Human Diseases	Cancers: Ov
5/159	686/28425	0.338900623	0.664379439	Environmental Information	Signal trans
1/159	75/28425	0.343775893	0.667329674	Metabolism	Carbohydra
1/159	77/28425	0.351116201	0.674961241	Organismal Systems	Excretory s
1/159	79/28425	0.358374914	0.682290701	Organismal Systems	Endocrine s
2/159	226/28425	0.361064274	0.68086406	Organismal Systems	Circulatory
1/159	82/28425	0.36911196	0.689473283	Metabolism	Lipid metal
3/159	408/28425	0.399980745	0.740151285	Genetic Information Proces	Folding, so
1/159	91/28425	0.40026347	0.733816362	Environmental Information	Signal trans
1/159	92/28425	0.403628971	0.733197581	Human Diseases	Substance (
2/159	249/28425	0.406658684	0.731985632	Human Diseases	Cancers: Sp
1/159	95/28425	0.413613283	0.737796667	Environmental Information	Signal trans
4/159	591/28425	0.421839284	0.745751591	Cellular Processes	Cellular cor
3/159	423/28425	0.422395363	0.740126388	Human Diseases	Cancers: Ov
1/159	99/28425	0.426667764	0.741054537	Organismal Systems	Digestive sy
3/159	430/28425	0.432762006	0.74510328	Human Diseases	Infectious (
1/159	101/28425	0.433086289	0.739233493	Organismal Systems	Endocrine s
2/159	264/28425	0.435549886	0.737084422	Cellular Processes	Cell growth
1/159	102/28425	0.436268724	0.73204413	Organismal Systems	Endocrine s
2/159	266/28425	0.439345701	0.731012174	Organismal Systems	Immune sy
2/159	266/28425	0.439345701	0.731012174	Organismal Systems	Immune sy
1/159	103/28425	0.439433405	0.719072845	Metabolism	Lipid metal
1/159	105/28425	0.445709902	0.72336525	Organismal Systems	Digestive sy
1/159	108/28425	0.45499394	0.732429269	Human Diseases	Cancers: Sp
1/159	109/28425	0.45805415	0.731409045	Human Diseases	Cancers: Sp
1/159	109/28425	0.45805415	0.731409045	Metabolism	Carbohydra
1/159	113/28425	0.470125177	0.738768136	Organismal Systems	Digestive sy
2/159	284/28425	0.472867167	0.737225978	Organismal Systems	Immune sy
1/159	115/28425	0.476060117	0.736405493	Organismal Systems	Aging
4/159	638/28425	0.479838072	0.736495645	Human Diseases	Endocrine a
2/159	288/28425	0.480153431	0.73131061	Organismal Systems	Immune sy
1/159	117/28425	0.481928994	0.728411762	Metabolism	Amino acid
5/159	826/28425	0.492605755	0.738908633	Human Diseases	Cancers: Ov
1/159	121/28425	0.493471479	0.734641751	Human Diseases	Substance (
2/159	297/28425	0.496321556	0.733370658	Human Diseases	Immune di
1/159	124/28425	0.501960308	0.736208452	Human Diseases	Cancers: Sp
1/159	126/28425	0.507540835	0.738919746	Organismal Systems	Nervous sy
3/159	489/28425	0.517025705	0.74723423	Organismal Systems	Immune sy
1/159	133/28425	0.526587817	0.755539041	Metabolism	Lipid metal
2/159	316/28425	0.529392698	0.754098951	Organismal Systems	Immune sy
2/159	325/28425	0.544540322	0.770135599	Human Diseases	Substance (

1/159	143/28425	0.552534565	0.775899602	Metabolism	Metabolism
1/159	145/28425	0.557551768	0.777431338	Human Diseases	Cancers: Sp
1/159	146/28425	0.560039366	0.775439122	Organismal Systems	Endocrine s
3/159	522/28425	0.561173855	0.771614051	Cellular Processes	Transport a
2/159	340/28425	0.569029576	0.777019697	Genetic Information Proces	Folding, sor
3/159	533/28425	0.57534504	0.780262452	Environmental Information	Signal trans
2/159	357/28425	0.595626132	0.802271933	Human Diseases	Infectious d
1/159	161/28425	0.595727942	0.796987382	Organismal Systems	Immune sy
1/159	163/28425	0.600263718	0.79766588	Metabolism	Xenobiotics
1/159	170/28425	0.615744332	0.812782519	Human Diseases	Drug resist
3/159	582/28425	0.634930439	0.832557794	Human Diseases	Cardiovasc
1/159	180/28425	0.636831483	0.8295568	Human Diseases	Cancers: Sp
1/159	180/28425	0.636831483	0.8295568	Metabolism	Nucleotide
1/159	180/28425	0.636831483	0.8295568	Human Diseases	Infectious d
4/159	789/28425	0.64702212	0.826518579	Human Diseases	Neurodege
1/159	188/28425	0.652869915	0.828642584	Organismal Systems	Immune sy
3/159	602/28425	0.657533218	0.829245714	Cellular Processes	Cell growth
1/159	193/28425	0.662534436	0.830264673	Human Diseases	Cancers: Sp
1/159	208/28425	0.689952655	0.859186326	Human Diseases	Infectious d
1/159	208/28425	0.689952655	0.859186326	Genetic Information Proces	Translation
1/159	208/28425	0.689952655	0.859186326	Organismal Systems	Aging
1/159	213/28425	0.698590798	0.853833197	Environmental Information	Signal trans
1/159	218/28425	0.706989747	0.858797361	Metabolism	Carbohydra
2/159	440/28425	0.707897558	0.854656808	Human Diseases	Infectious d
1/159	220/28425	0.710283833	0.8523406	Organismal Systems	Immune sy
1/159	226/28425	0.719946906	0.858731852	Organismal Systems	Nervous sy
1/159	231/28425	0.727754312	0.862846429	Cellular Processes	Transport a
2/159	470/28425	0.741603718	0.874032953	Genetic Information Proces	Translation
3/159	693/28425	0.747637188	0.875929959	Environmental Information	Signal trans
1/159	251/28425	0.75687943	0.881541925	Environmental Information	Signal trans
2/159	493/28425	0.765158655	0.885973179	Organismal Systems	Endocrine s
3/159	714/28425	0.765534329	0.881254634	Human Diseases	Cancers: Sp
4/159	949/28425	0.781243902	0.89414042	Human Diseases	Neurodege
1/159	274/28425	0.786563722	0.89505527	Human Diseases	Infectious d
1/159	283/28425	0.797172888	0.901944181	Human Diseases	Infectious d
1/159	283/28425	0.797172888	0.901944181	Human Diseases	Infectious d
1/159	287/28425	0.801718253	0.896837368	Organismal Systems	Endocrine s
1/159	302/28425	0.817879863	0.909776477	Cellular Processes	Cell growth
1/159	320/28425	0.835554434	0.92424457	Human Diseases	Infectious d
1/159	326/28425	0.841058525	0.925164378	Organismal Systems	Sensory sys
1/159	327/28425	0.841957906	0.921036825	Metabolism	Nucleotide
2/159	631/28425	0.870852867	0.947411361	Cellular Processes	Transport a
2/159	639/28425	0.875392524	0.94714601	Human Diseases	Infectious d
3/159	887/28425	0.876791747	0.943504163	Metabolism	Energy met
1/159	377/28425	0.881030025	0.942940243	Human Diseases	Immune dis
1/159	388/28425	0.888243191	0.945549203	Organismal Systems	Circulatory
1/159	406/28425	0.899120768	0.952010224	Human Diseases	Infectious d

1/159	424/28425	0.908945611	0.957293782	Environmental Information	Signal trans
1/159	439/28425	0.916402191	0.960040391	Human Diseases	Infectious c
1/159	443/28425	0.918285976	0.956950649	Human Diseases	Infectious c
1/159	467/28425	0.928734886	0.96277229	Human Diseases	Infectious c
4/159	1314/2842	0.939537641	0.968898192	Organismal Systems	Environme
1/159	501/28425	0.941303584	0.965689687	Cellular Processes	Cell growth
2/159	827/28425	0.947733664	0.967274564	Cellular Processes	Transport a
1/159	623/28425	0.970798969	0.985734337	Human Diseases	Infectious c
2/159	958/28425	0.972205022	0.982125481	Organismal Systems	Sensory sys
1/159	686/28425	0.979662483	0.984635389	Human Diseases	Cardiovasc
2/159	2464/2842	0.999991498	0.999991498	Genetic Information Proces	Translation

:egory
ıolecules and interaction
stem
ɔecific types
system
stem
system
verview
mmunity - eukaryotes
verview
diseases: Viral
stem
ystem
system
diseases: Parasitic
stem
stem
ystem
system
sduction
sduction
stem
sduction
ıolecules and interaction
stem
verview
stem
ɔecific types
and metabolic diseases
system
sduction
sduction
stem
system
sduction
stem
sduction
ent
sduction
system
system
system
synthesis and metabolism
stem
stem
n of cofactors and vitamins
diseases: Parasitic

olecules and interaction
n of cofactors and vitamins
sduction
mmunity - eukaryotes
sduction
diseases: Viral
pecific types
verview
diseases: Bacterial
pecific types
synthesis and metabolism
pecific types
sduction
nerative diseases
diseases: Parasitic
y
pecific types
mmunity - eukaryotes
ntal adaptation
sduction
stem
system

ent
and metabolic diseases
sduction
synthesis and metabolism
diseases: Viral
sduction
bolism
ystem
ance: Antineoplastic
sduction
sduction
pecific types
system
ystem
s biodegradation and metabolism
ate metabolism
sduction
olecules and interaction
system
n of cofactors and vitamins
system
and metabolic diseases
ystem
ance: Antineoplastic

diseases: Viral
stem
dependence
sduction
ystem
and metabolic diseases
verview
sduction
ate metabolism
ystem
system
 system
polism
rting and degradation
sduction
dependence
pecific types
sduction
mmunity - eukaryotes
verview
ystem
diseases: Viral
system
n and death
system
stem
stem
polism
ystem
pecific types
pecific types
ate metabolism
ystem
stem

and metabolic diseases
stem
l metabolism
verview
dependence
seases
pecific types
stem
stem
polism
stem
dependence

n of cofactors and vitamins
pecific types
system
and catabolism
rting and degradation
sduction
diseases: Viral
stem
s biodegradation and metabolism
ance: Antineoplastic
ular diseases
pecific types
metabolism
diseases: Viral
nerative diseases
stem
n and death
pecific types
diseases: Parasitic
n

sduction
ate metabolism
diseases: Bacterial
stem
stem
and catabolism
n
sduction
sduction
system
pecific types
nerative diseases
diseases: Parasitic
diseases: Parasitic
diseases: Bacterial
system
n and death
diseases: Bacterial
stem
metabolism
and catabolism
diseases: Viral
tabolism
seases
system
diseases: Bacterial

sduction
diseases: Bacterial
diseases: Viral
diseases: Bacterial
ntal adaptation
n and death
and catabolism
diseases: Viral
stem
ular diseases
[illegible]
